# ZnO Quantum Dots@CsPbBr_3_ Poly‐Heterocrystalline Film Enables High‐Performance Floating‐Gate Transistor Arrays for Edge Computing

**DOI:** 10.1002/advs.202506357

**Published:** 2025-08-30

**Authors:** Jiajun Xu, Bo Tong, Nian Dai, Tongjian Liu, Zhibo Liu, Dingdong Zhang, Yan Liang, Song Qiu, Lai‐Peng Ma, Jinhong Du

**Affiliations:** ^1^ Shenyang National Laboratory for Materials Science Institute of Metal Research Chinese Academy of Sciences Shenyang 110016 China; ^2^ School of Materials Science and Engineering University of Science and Technology of China Shenyang 110016 China; ^3^ Advanced Materials Division Suzhou Institute of Nano‐Tech and Nano‐Bionics Chinese Academy of Sciences Suzhou 215123 China

**Keywords:** edge computing, floating‐gate photosensitive transistor, optoelectronic synapses, poly‐heterocrystalline film, ZnO quantum dots @ CsPbBr_3_

## Abstract

Nonvolatile optoelectronic synapses motivated by the human eye can effectively function as convolutional kernels to preprocess images, demonstrating significant promise for edge computing. Among the optoelectronic synapses, the floating‐gate photosensitive transistor (FG‐PT) is particularly noteworthy due to its rapid response speed and excellent retention. Although some FG‐PTs are reported, they still suffer from high operating voltages, low conductance ratios, and difficulties in array preparation. Here, a ZnO QDs@CsPbBr_3_ poly‐heterocrystalline (PHC) film to serve as the floating gate layer of FG‐PT is synthesized. The PHC film combines the desirable properties of each phase, exhibiting both high electrical conductivity and excellent photoelectric properties. The high electrical conductivity allows FG‐PT to store a large amount of charge at a low voltage (1 V). While the excellent photoelectric properties facilitate the gradual erasure of these charges under light pulses, resulting in a large conductance ratio (≈3.57×10^5^). Moreover, the excellent film‐forming properties of the PHC film enable the fabrication of an FG‐PT array comprising 25,600 devices on 1 cm^2^ substrate. Using the FG‐PT array to preprocess images achieves a significantly higher recognition precision at the fifth epoch (87.64%) than that of the original images (58.08%) and CMOS‐processed images (77.68%), indicating great potential for edge computing.

## Graphical abstract

The ZnO QDs@CsPbBr_3_ poly‐heterocrystalline film, featuring a semi‐coherent interface, delivers high electrical conductivity and excellent photoelectric properties. Functioning as a floating gate in photosensitive transistors, it enables high hole storage at 1 V and achieves a conductivity ratio of ≈3.57×10^5^. A high‐density array of 25,600 devices (145 ppi) is fabricated, demonstrating a significantly higher recognition precision (87.64%) at the fifth epoch than that of the original images (58.08%) and CMOS‐processed images (77.68%), rivaling CMOS circuits for edge computing applications.

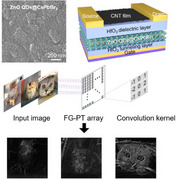

## 1 Introduction

Edge computing is a process that extracts image feature information (image preprocessing) on noncloud devices, significantly enhancing the computing efficiency of neural networks.^[^ ^1^, ^2^ ^]^ Optoelectronic synapses, inspired by the human eye, can serve as noncloud devices for image preprocessing.^[^ ^3^, ^4^, ^5^ ^]^ Nonvolatile optoelectronic synapses can act as convolution kernels to extract image feature information, which is flexible and suitable for a variety of tasks.^[^ ^6^ ^]^ Among the various nonvolatile optoelectronic synapses, the floating‐gate photosensitive transistor (FG‐PT) is particularly noteworthy due to its rapid response speed and excellent retention.^[^ ^7^ ^]^ Although some FG‐PTs have been developed using organic semiconductors, perovskites, and quantum dots (QDs) as FG layer, the limited electrical conductivity and photoelectric properties of these materials result in high operating voltages (>10 V) and low conductance ratios (<10^4^) of FG ‐PTs.^[^ ^6^, ^7^, ^8^, ^9^, ^10^, ^11^, ^12^, ^13^ ^]^ The high operating voltage leads to increased power consumption, while the low conductance ratio restricts the range of weight selection of convolution kernel, significantly affecting the precision of image feature extraction.^[^ ^14^, ^15^, ^16^ ^]^ In addition, their large‐scale integration poses challenges. The number of integrated FG‐PTs is generally fewer than 10, and the resolution is significantly lower than the human eye, further impeding their practical uses.^[^ ^17^, ^18^, ^19^ ^]^

**Figure 1:**
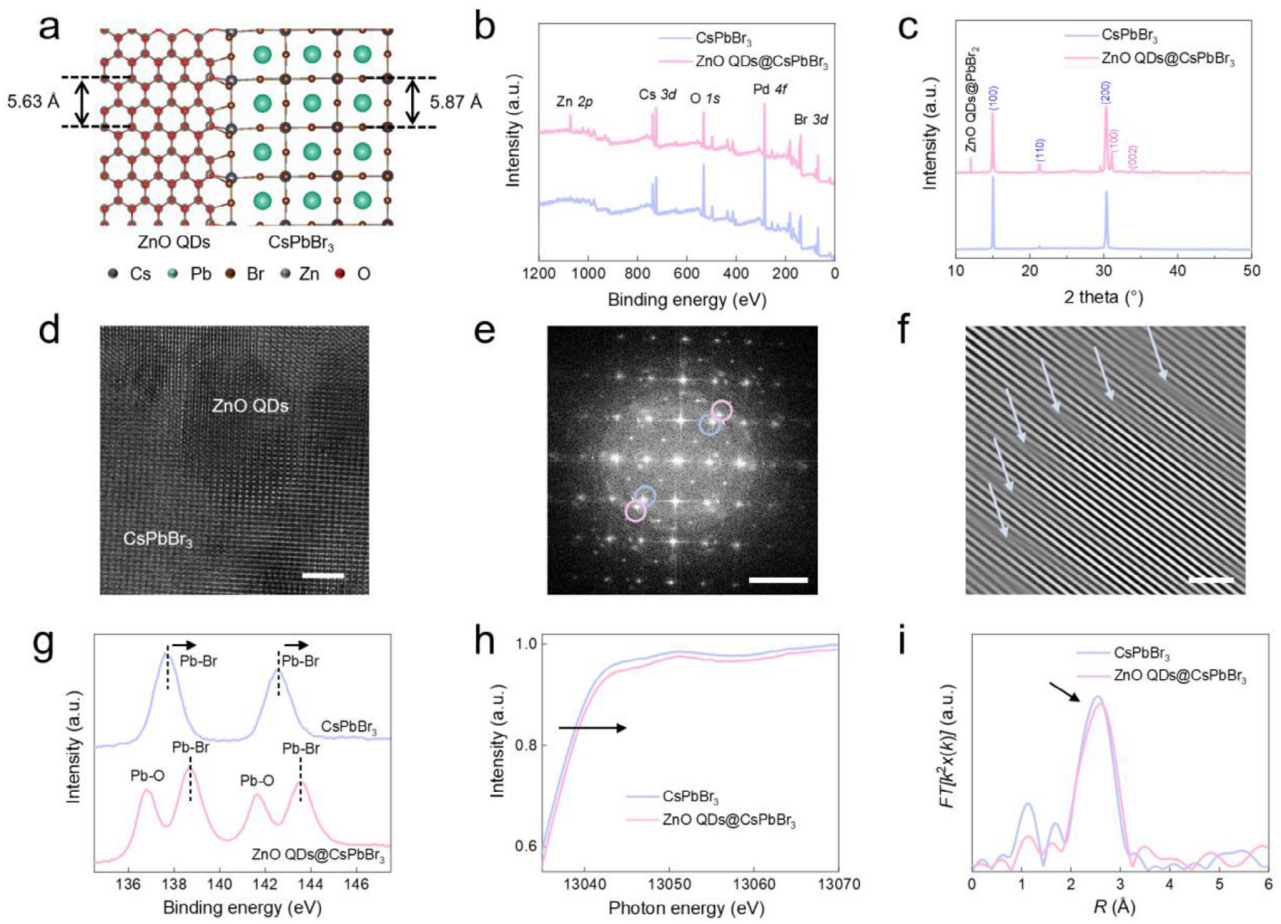
Structure of ZnO QDs@CsPbBr_3_ PHC and the formation of Pb─O bond. a) Schematic diagram of ZnO QDs@CsPbBr_3_ PHC. b) Full XPS spectra of CsPbBr_3_ and ZnO QDs@CsPbBr_3_ PHC. c) XRD patterns of CsPbBr_3_ and ZnO QDs@CsPbBr_3_ PHC. The XRD pattern of ZnO QDs@CsPbBr_3_ PHC consists of both CsPbBr_3_ (blue) and ZnO QDs (pink) characteristic peaks. d) HAADF ‐STEM images of ZnO QDs@CsPbBr_3_ PHC. Scale bar, 5 nm. e) The corresponding FFTs. The mass fraction of ZnO QDs in ZnO QDs@CsPbBr_3_ PHC is 2.4%. Scale bar, 2 nm^−1^. The pink circles represent ZnO QDs, while the blue circles represent CsPbBr_3_. f) IFFT image of ZnO QDs@CsPbBr_3_ PHC, showing only a small number of interfacial defects and lattice distortion. Scale bar, 2 nm. g) Pb 4f XPS spectra of CsPbBr_3_ and ZnO QDs@CsPbBr_3_ PHC films. h) Normalized Pb K‐edge XAFS spectra of CsPbBr_3_ and ZnO QDs@CsPbBr_3_. i) The corresponding Fourier transform of Pb K‐edge XAFS spectra shown in Figure 1h.

Poly‐heterocrystalline (PHC) films can be fabricated from two semiconductors with similar crystal structures using solution processing.^[^ ^20^, ^21^, ^22^, ^23^ ^]^ Unlike the mechanical mixing of two semiconductors, PHC films typically have good lattice matching between the components, therefore fully integrate and leverage the superior properties of each component.^[^ ^24^, ^25^, ^26^, ^27^ ^]^ CsPbBr_3_ is a semiconductor known for its excellent optoelectronic properties, which have facilitated the development of high‐performance optoelectronic devices, including photodetectors and solution‐processed light‐emitting diodes with high efficiency.^[^ ^28^, ^29^, ^30^, ^31^, ^32^ ^]^ ZnO QDs are semiconductor materials with excellent conductivity.^[^ ^33^, ^34^ ^]^ Moreover, CsPbBr_3_ and ZnO QDs possess related crystal structures (tetragonal and rock‐salt, respectively). The (1‐10) plane of ZnO QDs and the (200) plane of CsPbBr_3_ have similar lattice constant with only 4.3% mismatch (**Figure** **1**a), laying a solid foundation for the fabrication of ZnO QDs@CsPbBr_3_ PHC film that combines excellent photoelectric properties of CsPbBr_3_ and high electrical conductivity of ZnO QDs.

Here, we synthesized ZnO QDs@CsPbBr_3_ PHC with a semi‐coherent interface between the ZnO QDs and CsPbBr_3_ using a sol‐gel strategy and further used the PHC film as the FG layer to construct FG‐PT.^[^ ^35^ ^]^ In the semi‐coherent interface, a Pb‐O coordination bond is formed, which facilitates hole transfer from ZnO QDs to CsPbBr_3_, thereby enhancing the electrical conductivity and photoelectric properties of CsPbBr_3_. The high electrical conductivity of the PHC film enables FG‐PT to store a large amount of holes at low voltage (1 V) with large storage density (1.18×10^13^ cm^−2^). While the excellent photoelectric properties facilitate numerous stored holes being erased under light pulses, resulting in a large conductivity ratio (≈3.57×10^5^). Moreover, the excellent film formation properties of PHC enable the fabrication of FG‐PT arrays comprising 25,600 devices on 1 cm^2^ substrate, which is comparable to the resolution of the human eye. We further employed the FG‐PT arrays to act as convolution kernel to extract image features, which shows a significantly higher recognition precision (87.64%) at the fifth epoch than that of the original images (58.08%) and CMOS‐processed images (77.68%), indicating great potential for edge computing.

## 2 Results

We developed a sol‐gel strategy to synthesize ZnO QDs@CsPbBr_3_ PHC and further obtain PHC film by one‐step spin coating (Figure S1, Supporting Information). In detail, PbBr_2_ was first mixed into ZnO QDs in Dimethylsulfoxide (DMSO) solvent to form ZnO QDs@PbBr_2_ colloid solution (Figure S1a, Supporting Information). ZnO QDs diffraction peaks appearing and the peaks of PbBr_2_ shift in X‐ray diffraction (XRD) pattern confirm the formation of ZnO QDs@PbBr_2_ (Figure S2, Supporting Information). Next, CsBr was added into the ZnO QDs@PbBr_2_ colloid solution and heated at 60 °C for 6 h. During this process, Cs^+^ could occupy the octahedral sites of ZnO QDs@PbBr_2_ colloid to form ZnO QDs@CsPbBr_3_ PHC colloid solution (Figure S1b, Supporting Information). Finally, ZnO QDs@CsPbBr_3_ PHC film (Figure S1c, Supporting Information) was obtained by one‐step spin ‐coating followed by heating at 60 °C for 20 min. X ‐ray Photoelectron Spectroscopy (XPS), XRD and Energy‐dispersive X‐ray spectroscopy (EDS) results demonstrate that ZnO QDs have been successfully integrated with CsPbBr_3_ (Figure 1b,c; Figure S2b,c and Table S1, Supporting Information).

Density Functional Theory (DFT) calculations reveal that the (1‐10) plane of ZnO QDs and the (200) plane of CsPbBr_3_ have similar lattice constant with only 4.3% mismatch (Figure 1a). Atomic‐level high‐angle annular dark‐field scanning transmission electron microscopy (HAADF‐STEM) was utilized to characterize the semi‐coherent interface of ZnO QDs@CsPbBr_3_ (Figure 1d). Consistent with the calculation result, the lattice fringes of CsPbBr_3_ align well with ZnO QDs. Fast Fourier Transform (FFT) of the ZnO QDs@CsPbBr_3_ PHC (Figure 1e) displays diffraction spots of both CsPbBr_3_ and ZnO QDs. The angular difference between their diffraction spots is less than 5°, confirming a good alignment between them. By selecting the FFT point of CsPbBr_3_ and ZnO QDs in Figure 1e, the Inverse Fast Fourier Transform (IFFT) image was obtained, which reveals only a small number of interfacial defects and lattice distortion, possibly due to the strain caused by the different elastic modulus of ZnO QDs and CsPbBr_3_ (Figure 1f). Furthermore, the analysis of five additional distinct areas, including their corresponding FFT/IFFT patterns, further corroborates the excellent lattice alignment between ZnO QDs and CsPbBr_3_, validating the uniformity and reproducibility of this semi‐coherent structure in the ZnO QDs@CsPbBr_3_ PHC (Figure S3, Supporting Information).

Besides the semi‐coherent interface, a Pb‐O bond is formed between ZnO QDs and CsPbBr_3_. The Pb 4f XPS spectrum shows the presence of both Pb‐Br and Pb‐O bonds in ZnO QDs@CsPbBr_3_ PHC (Figure 1g). Compared with CsPbBr_3_, Pb 4f exhibits a 0.7 eV blueshift while the O 1s shift 0.5 eV to the opposite direction (Figure S4, Supporting Information), indicating the coordination between Pb of CsPbBr_3_ and O of ZnO QDs. Pd K‐edge X‐ray Absorption Fine Structure (XAFS) spectra further confirmed the formation of Pb‐O bond (Figure 1h; Table S2, Supporting Information). The real‐space transformation and wavelet analysis indicate that the strongest peak in XAFS spectra of ZnO QDs@CsPbBr_3_ PHC corresponds to the Pb‐Br bond, with an increased bond length compared to that of CsPbBr_3_ (Figure 1i). Additionally, the coordination number of the Pb‐Br bond in ZnO QDs@CsPbBr_3_ PHC is significantly larger than that of CsPbBr_3_, and it is closer to the coordination number of the Pb‐O bond. This suggests a coordination interaction between the Pb in CsPbBr_3_ and the O in ZnO QDs. The semi‐coherent interface and Pb‐O bond formation allow ZnO QDs to be embedded in the CsPbBr_3_ lattice to achieve ZnO QDs@CsPbBr_3_ PHC.

**Figure 2:**
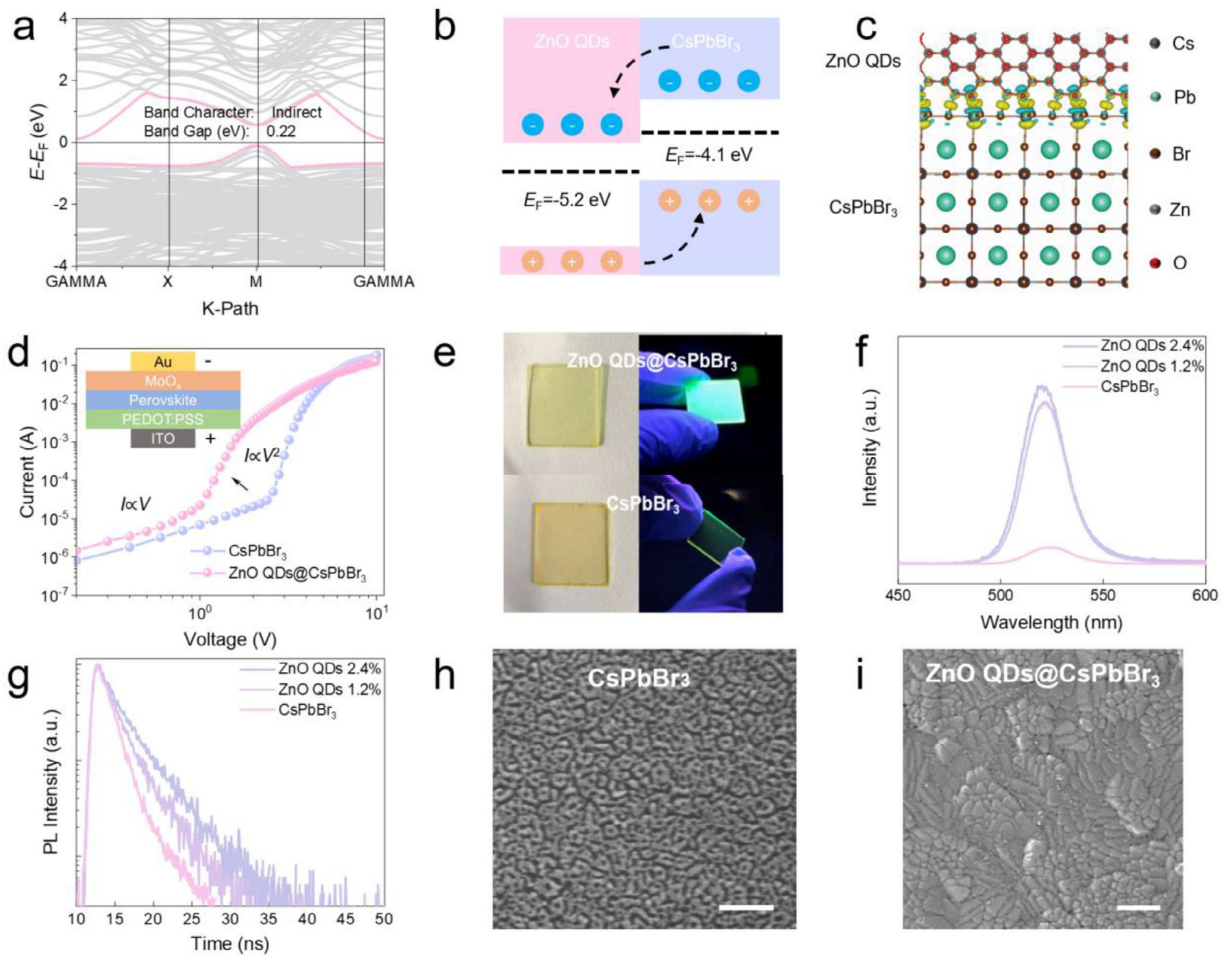
Optoelectronic and film‐forming properties of ZnO QDs@CsPbBr_3_ PHC. a) The DFT calculated band energy alignment of the ZnO QDs@CsPbBr_3_ PHC interface, indicating a small band gap of 0.22 eV. b) The band alignment of CsPbBr_3_ and ZnO QDs. The valence band maximum (VBM) and Conduction Band Minimum (CBM) are estimated as −5.8 and −3.5 eV for CsPbBr_3_,^[^ ^36^ ^]^ respectively, while they are −8.2 and −4.3 eV for ZnO QDs.^[^ ^37^ ^]^ c) Bader differential charge density of ZnO QDs@CsPbBr_3_ PHC, cyan indicates electron loss in CsPbBr_3_, and yellow signifies electron gain. d) The *I–V* curves of CsPbBr_3_ and ZnO QDs@CsPbBr_3_ PHC films measured using the SCLC method. Inset is the schematic structure of a hole‐only device. e) Photograph of ZnO QDs@CsPbBr_3_ PHC and CsPbBr_3_ films under natural light (left) and 305 nm light (right). The size of the PHC film is 2 cm × 2 cm. f) PL spectra and g) Time‐resolved photoluminescence (TRPL) curves of CsPbBr_3_ and ZnO QDs@CsPbBr_3_ PHC with different mass fractions of ZnO QDs. h) SEM images of CsPbBr_3_ and i) ZnO QDs@CsPbBr_3_ PHC films with mass fraction of 2.4%. Scale bars, 1 µm and 200 nm, respectively.

The formation of Pb‐O coordination bond reduces the density of interface defects and lowers the charge transfer barrier. DFT calculations revealed that the semi‐coherent interface between the (1‐10) plane of ZnO QDs and the (200) plane of CsPbBr_3_ has a very small bandgap of 0.22 eV, facilitating charge transfer between ZnO QDs and CsPbBr_3_ at interface (**Figure** **2**a). Therefore, holes can easily transfer from ZnO QDs to CsPbBr_3_, thereby achieving p‐type doping of CsPbBr_3_ (Figure 2b). Differential charge density analysis supports this charge transfer process, showing that CsPbBr_3_ lose electrons while ZnO QDs gains them (Figure 2c). Ultraviolet photoelectron spectroscopy (UPS) reveals that, compared to pristine CsPbBr_3_, the introduction of ZnO QDs results in a downward shift in the Fermi level (from −5.0 to −5.2 eV) and the valence band maximum (VBM) (from −5.9 to −6.8 eV) in the ZnO QDs@CsPbBr_3_ PHC as the mass fraction of ZnO QDs increases (Figure S5, Supporting Information).

The effective p‐type doping of ZnO QDs in CsPbBr_3_ could enhance both electrical conductivity and photoelectric properties of PHC. We constructed hole‐only devices with a configuration of ITO/PEDOT:PSS/perovskite/MoO_3_/Au to measure the mobility of ZnO QDs@CsPbBr_3_ PHC films using the Space‐Charge‐Limited Current (SCLC) method (Figure 2d). By fitting the I‐V curve with the Mott–Gurney law:

(1) $$I=\frac{9\varepsilon \varepsilon_{0}\mu SV^{2}}{8L^{3}}$$

where ε is the relative permittivity constant, ε_0_ is the vacuum permittivity, μ is the carrier mobility, L is the distance between the electrodes, and S is the cross‐sectional area of the device. The measured hole mobility for the ZnO QDs@CsPbBr_3_ PHC film with ZnO content of 2.4% was 9.5 cm^2^ V^−1^ s^−1^, which is three times higher than that of the CsPbBr_3_ film (3.1 cm^2^ V^−1^ s^−1^). Furthermore, both the photoluminescence (PL) intensity and exciton lifetime of the ZnO QDs@CsPbBr_3_ PHC films increased significantly (Figure 2e–g; Note S1, Supporting Information). Additionally, the photocurrent and light‐to‐dark current ratio of the ZnO QDs@CsPbBr_3_ PHC films were notably higher than those of the CsPbBr_3_ films (Figure S6, Supporting Information). These results demonstrate the enhanced photoelectric properties of the ZnO QDs@CsPbBr_3_ PHC films.

The formation of Pb‐O bond could also improve the film‐forming properties of ZnO QDs@CsPbBr_3_ PHC. The limited solubility of CsBr in the precursor solutions, such as DMSO, often leads to a not very dense CsPbBr_3_ film with a rough surface (Figure 2h; Figure S7a, Supporting Information). The introduction of ZnO QDs into the precursor solution initiates the formation of large pre‐nucleation clusters through coordination with PbBr_2_ (Figure S2, Supporting Information). These ZnO QDs@PbBr_2_ pre‐nucleation clusters act as both building blocks and nucleation sites, aiding the dissolution of CsBr in DMSO. Therefore, the ZnO QDs@CsPbBr_3_ PHC film exhibits smaller grain sizes compared to the pristine CsPbBr_3_ film (Figure 2i; Figure S7b, Supporting Information). Additionally, it has lower film roughness, and the grains are more efficiently packed into a dense film without the presence of pinholes. We conducted PL mapping over a 20 µm×20 µm area, showing nearly uniform PL intensity, further confirming the uniformity of the ZnO QDs@CsPbBr_3_ PHC film (Figure S7c, Supporting Information). Grazing‐Incidence Wide‐Angle X‐ray Scattering (GIWAXS) measurements demonstrate that ZnO QDs@CsPbBr_3_ PHC film has significantly improved crystallographic orientation (Figure S8, Supporting Information). More importantly, the ZnO QDs@CsPbBr_3_ PHC film exhibits superior ambient stability compared to pristine CsPbBr_3_, with its PL intensity showing minimal degradation even after one month of exposure to ambient conditions (Figure 2e; Figure S9, Supporting Information).

**Figure 3:**
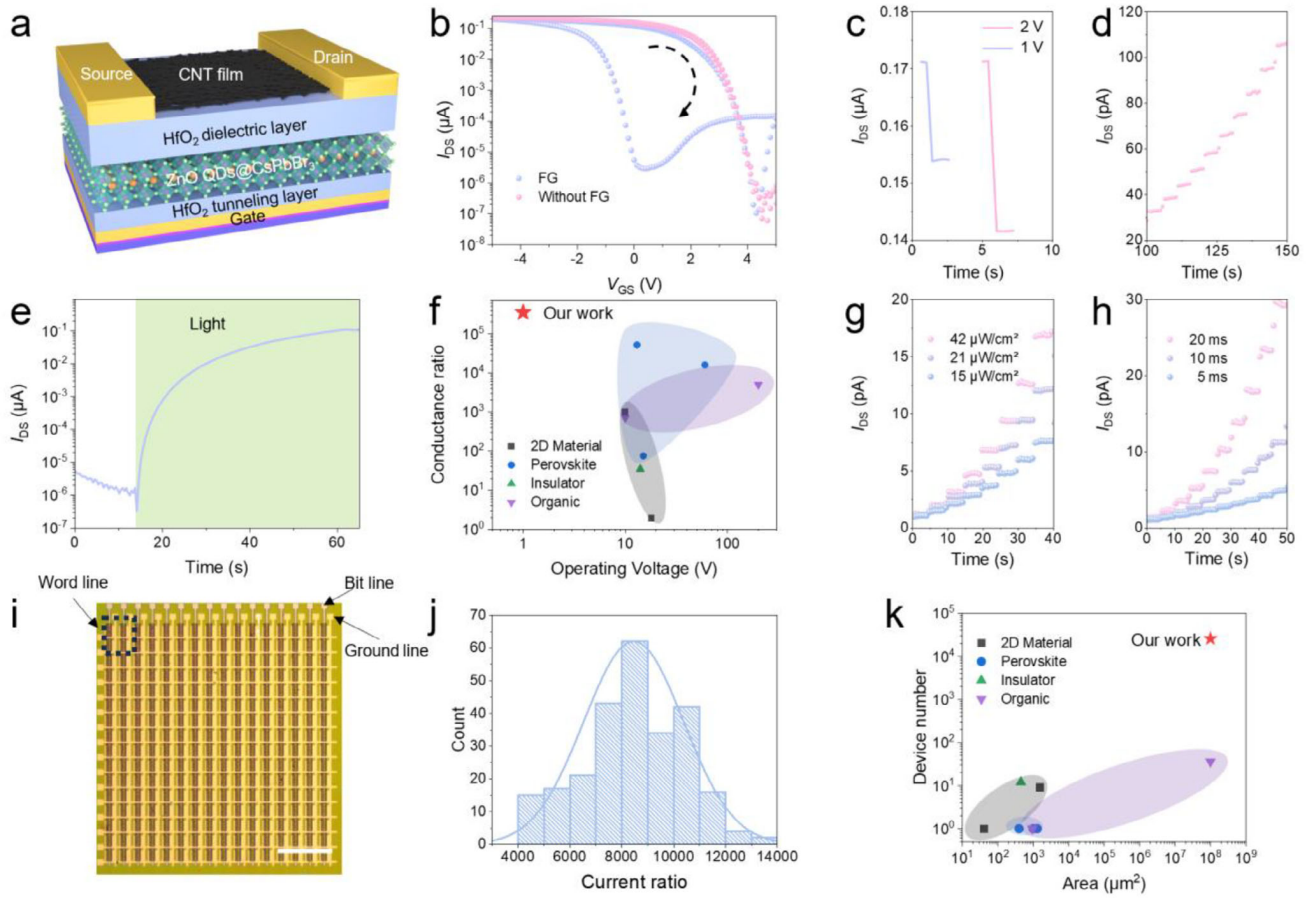
The working mechanism, synaptic behavior and array integration of FG‐PT. a) Schematic diagram of the FG‐PT device. b) Transfer curves of the FG‐PT device in the dark, with and without the FG layer. The FG layer induces clockwise hysteresis of the FG‐PT device. c) The electrical writing process of the FG‐PT device. The pulse widths are 400 ms. Prior to applying positive gate voltages, a negative *V*_GS_ pulse (−5 V, 300 ms) was applied to fully erase holes stored in the floating gate layer. d) The light erasing process of FG‐PT device. Each pulse could erase partial holes stored in the FG layer, resulting in a gradual increase of device conductance. The wavelength of the light is 516 nm, the pulse width is 10 ms, the pulse interval is 5 s, and the light intensity is 21 µW cm^−2^. e) The transition of the FG‐PT device from HRS to LRS under a 516 nm laser, showing a high conductance ratio of ≈3.57 × 10^5^. The light intensity is 21 µW cm^−2^. f) Comparison of the conductance ratio and operating voltage of our devices with the reported FG‐PT. g) Modulation of the synaptic weight of FG‐PT under 516 nm laser with different light intensities. The light pulse width is 10 ms. h) Modulation of the synaptic weight of FG‐PT under a 516 nm laser with different light pulse widths. The light intensity is 21 µW cm^−2^. i) The optical image of a high‐density FG‐PT array. The word line is connected to the drain, the bit line is connected to the gate, and the ground line is connected to the source. Scale bar, 200 µm. A 15 nm‐thick HfO_2_ layer was integrated into the device array to avoid short circuit issues and suppress the crosstalk. j) Statistics of the current ratio at *V*_GS_ = 2 V of 256 devices in the FG‐PT array in the dark. *V*_DS_ = 100 mV. k) Comparison of the area and device number of our device array with the reported FG‐PT array. See Table S3 (Supporting Information) for details.

We further constructed the FG‐PT devices using ZnO QDs@CsPbBr_3_ PHC as the FG layer and carbon nanotubes (CNTs) as the channel layer (**Figure** **3**a; Figure S10, Supporting Information). Note that CNTs have negligible photo‐response (Figure S11, Supporting Information), which effectively prevents information crosstalk. We first measured the transfer curve of the FG‐PT in the dark (Figure 3b). Compared to the device without FG, the transfer curve of the FG‐PTs demonstrates a significant hysteresis, indicating holes can be written into the ZnO QDs@CsPbBr_3_ PHC under positive gate voltage (Figures S12a and S13a, Supporting Information). The high electrical conductivity of the ZnO QDs@CsPbBr_3_ PHC allows it to store many holes at low operating voltages (down to 1 V) (Figure 3c; Figure S14a, Supporting Information), which results in ultralow writing power consumption of 178 fJ (Note S2 and Figure S15, Supporting Information). Moreover, devices using ZnO QDs@CsPbBr_3_ PHC as the floating gate layer exhibit significantly larger hysteresis and higher charge storage density (≈1.18 × 10^13^ cm^−2^) compared to FG‐PTs employing ZnO QDs and CsPbBr_3_ separately as the floating gate layer. This indicates that the formation of the PHC structure substantially improves the device's charge storage capability (Note S3 and Figure S16, Supporting Information).

After applying a light pulse, the holes stored in ZnO QDs@CsPbBr_3_ PHC could absorb photon energy, allowing them to overcome the interface barrier between the ZnO QDs@CsPbBr_3_ PHC and the HfO_2_ layer (Figure S12b, Supporting Information). Consequently, the holes can be erased from the ZnO QDs@CsPbBr_3_ PHC, which increases the conductance of the FG‐PT (Figure 3d,e; Figure S13b, Supporting Information). Each pulse can erase a portion of the holes, gradually increase the conductance of the FG‐PT and generate multiple resistance states, demonstrating excellent long‐term potentiation (LTP) behavior (Figure 3d; Figure S17, Supporting Information). Besides LTP behavior under light pulses, the device can write/erase holes using gate voltage, enabling it to exhibit long‐term depression and LTP behavior (Figures S14b, S18, and S19, Supporting Information). Due to the improved conductivity and photoelectronic properties of the PHC, light enables the numerous stored holes to absorb photon energy and be erased from the floating‐gate layer, resulting in a high conductance ratio of ≈3.57 × 10^5^ (Figure 3e). Note that the conductance ratio of device with PHC floating layer is significantly greater than that of the device utilizing the ZnO QDs and CsPbBr_3_ floating gate layer (Figure S20, Supporting Information). The high conductance ratio ensures that the device has a wider dynamic range to enrich the range of weight selection in convolution kernel, improving the precision of image feature extraction. Meanwhile, the device exhibits a fast response time as low as 10 ms (Figure S21, Supporting Information). Notably, the operating voltage and conductance ratio are the best among the FG‐PTs constructed with other FG such as 2D material, insulator, organic and perovskite in the references (Figure 3f; Table S3, Supporting Information).

Furthermore, the FG‐PT demonstrates good retention characteristics, with the retention time of both high‐resistance state (HRS) and low‐resistance state (LRS) exceeding 1000 s (Figure S22a, Supporting Information). Notably, increasing the thickness of HfO_2_ can further enhance the device's retention performance (Figure S22b, Supporting Information). The FG‐PT also exhibits good cyclic stability, showing no significant degradation after 1000 cycles (Figure S22c, Supporting Information). In addition, the synaptic weight of the FG‐PT device can be adjusted by varying the light intensity and pulse width (Figure 3g,h; Figure S23, Supporting Information). As the light intensity and pulse width increase, each light pulse can remove more holes, thus increasing the synaptic weight, which favors for the construction of various neural networks. Moreover, the exceptionally high quality of the ZnO QDs@CsPbBr_3_ PHC film as well as CNT and HfO_2_ layers (Figure S24, Supporting Information) allows us to fabricate FG‐PT array integrating 25,600 devices on 1 cm^2^ substrate (145 ppi), which is comparable to the resolution of the human eye (Figure 3i; Figure S25, Supporting Information). FG‐PTs in the array exhibit no short circuits, and the crosstalk between adjacent devices is negligible (Note S4, Figures S26 and S27, Supporting Information). Notably, the FG‐PT array demonstrates excellent uniformity. A random selection of 256 devices in the array functioned normally, with all devices exhibiting similar transfer characteristic curves and nearly identical current ratio (Figure 3j; Figure S28, Supporting Information). Compared to reported device arrays based on 2D material, insulator, organic and perovskite FG layer, our FG‐PT array offers significant advantages in terms of area and device count (Figure 3k; Table S3, Supporting Information).

**Figure 4:**
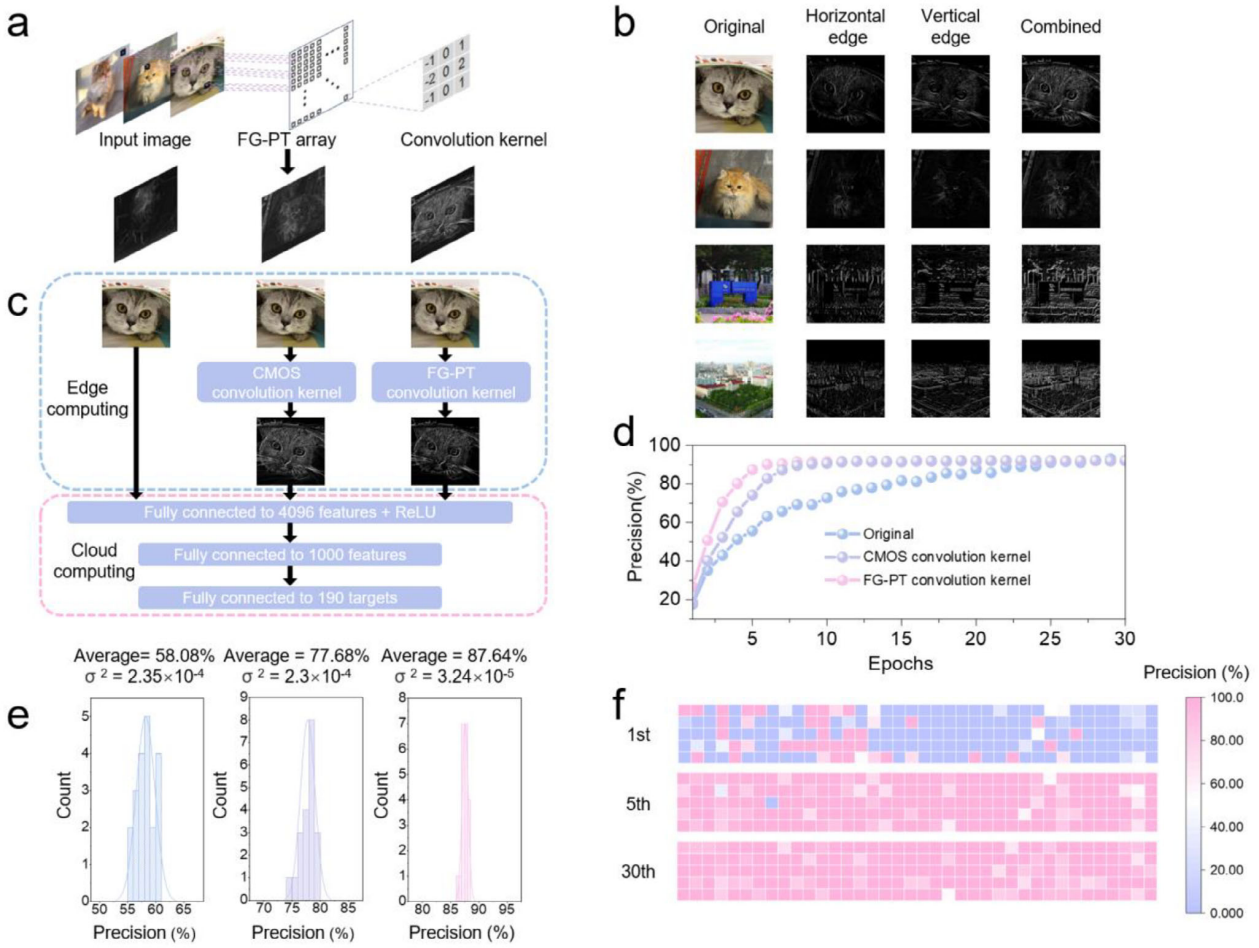
Simulation of the image feature extraction using FG‐PT array. a) The convolution operation applied to the input image. Convolutional kernels (gray boxes) at various positions enable simultaneous convolution of multiple images using the FG‐PT array. b) The original input images and the output images after convolution using the FG‐PT array as the convolution kernel. c) A model for cat face image classification. Both the original images and images processed by CMOS convolution kernels and FG‐PT convolution kernels are fed into the ANN network. Image feature extraction is performed as edge computing, while the ANN network functions as cloud computing. d) The dependence of recognition precision on epochs for different convolution kernels. e) The precision distribution of images obtained from original images (left), images processed with a CMOS convolution kernel (middle), and FG‐PT convolution kernel (right) at the 5th epoch. f) The recognition precision distribution of 190 cats at the 1st, 5th, and 30th epochs. Each differently colored square represents the recognition precision of a cat in the respective epoch.

The large‐area, uniform FG‐PT array paves the way for further high‐density integration, providing a solid foundation for its use in image preprocessing. Edge computing is a strategy that involves preprocessing images on noncloud devices before transmitting the data to the cloud for more complex processing using neural networks, thereby alleviating the load on cloud servers. Nonvolatile optoelectronic synapses can function as convolutional kernels, enabling a flexible approach to extracting a variety of image features. The FG‐PT array, based on ZnO QDs@CsPbBr_3_ PHC film, can serve as a noncloud device in edge computing due to its wide dynamic range (large conductance ratio), nonvolatile storage, and capability for large‐area integration. The large conductance ratio of the FG‐PT allows for flexible adjustment of the weights of the convolutional kernels to optimize feature extraction, ensuring high precision in edge computing. Moreover, our FG‐PT array comprises 2844 convolutional kernels, with each 3×3 group of devices forming a single kernel. Consequently, we can utilize these kernels to simultaneously extract features from 2844 original images, thereby enhancing the efficiency of edge computing (**Figure** **4**a). The processed images can then be transmitted to cloud‐based Artificial Neural Networks (ANNs) for image recognition.

We used images of animals and landscapes as examples to demonstrate the functionality of the FG‐PT array for edge computing. First, FG‐PT array captured both horizontal and vertical features, which were then combined to generate comprehensive edge feature images (Figure 4b). Next, the edge feature images processed by the FG‐PT array were transmitted to a cloud‐based ANN for recognition (Figure 4c). The performance evaluation revealed that the FG‐PT processed images could achieve a precision of 91% within 8 epochs (Figure 4d). To avoid the randomness of a single training cycle, we repeated the experiment 20 times and statistically analyzed the precision at the fifth epoch. The results show that images processed by the FG‐PT array achieve a precision of 87.64%, significantly higher than that of the original images (58.08%) and CMOS‐processed images (77.68%) (Figure 4e). In addition, we used the evaluation method of the confusion matrix, which provides a more comprehensive assessment of model performance across all classes rather than relying solely on overall accuracy. Simultaneous improvement in accuracy, precision, recall, and F1 score indicates balanced optimization (Note S5 and Figure S29, Supporting Information). Finally, we analyzed the precision throughout the network training process, observing a gradual improvement in the recognition and prediction of images of 190 cats at the 1st, 5th, and 30th epochs (Figure 4f). To further validate the universality of image feature extraction using the FG‐PT array, we conducted experiments employing the “Prewitt convolution kernel”, which yielded consistent results (Figure S30, Supporting Information). The images processed by the FG‐PT array achieved higher recognition precision. These results confirm the effective image preprocessing capabilities of the FG‐PT array, which not only enhances the computational efficiency and precision of cloud servers but also accelerates the training process of neural networks.

## 3 Conclusion

In summary, we have developed a novel ZnO QDs@CsPbBr_3_ PHC, which has a superior high electrical conductivity and photoelectric properties and excellent film forming properties to pristine CsPbBr_3_. By using ZnO QDs@CsPbBr_3_ PHC film as FG, we successfully fabricate a large area FG‐PT array with the integration density of 145 ppi, which is nearly equivalent to the resolution of the human eye. Moreover, the FG‐PT could achieve a large conductance ratio of 3.57×10^5^ and a low operate voltage of 1 V, both of which are the best among the FG‐PTs. We further perform image recognition and edge computing using the FG‐PT array. The results demonstrate the exceptional capability of image convolution preprocessing, offering a method for image processing on edge devices and significantly accelerating the training process of cloud‐based neural networks.

## 4 Experimental Section

### Preparation and Characterization of ZnO QDs@CsPbBr_3_ PHC Film

PbBr_2_ was first mixed with ZnO QDs in DMSO solvent to form ZnO QDs@PbBr_2_ colloid solution. Then, CsBr was added into the ZnO QDs@PbBr_2_ colloid solution and heated at 60 °C for 6 h to form ZnO QDs@CsPbBr_3_ colloid solution. Finally, ZnO QDs@CsPbBr_3_ PHC film was obtained by spin‐coating followed by heating at 60 °C for 20 min.

XPS (ESCALAB 250 instrument with Al Kα and He I radiation sources) were used to characterize the element compositions of ZnO QDs@CsPbBr_3_. The XPS spectra were fitted using the XPS peak 4.1 software in which a Shirley background was assumed. The structure of bulk ZnO QDs@CsPbBr_3_ crystals was characterized by XRD (Rigaku, D/MAX 2400 using Cu Kα radiation). HAADF‐STEM imaging and EDS analysis were performed on a FEI Titan Cube Themis G2 300 instrument equipped with a high‐brightness field‐emission gun (X‐FEG), double spherical aberration corrector, and a monochromator. The images were obtained at 300 kV. The morphology and thickness of ZnO QDs@CsPbBr_3_ film were characterized by an optical microscope (Nikon Eclipse LV100ND). The XAFS spectra were measured to explore the Pb‐O coordination bond in ZnO QDs@CsPbBr_3_ PHC. The Pb K‐edge XAFS data were collected at the BL14W1 station in Shanghai Synchrotron Radiation Facility (SSRF) in transition mode. The X ray was monochromatized by a double‐crystal Si (311) monochromator for SSRF. The monochromator was detuned to reject higher harmonics. The acquired XAFS data were processed and analyzed according to the standard procedures by using the WinXAS3.1 program. PL and TRPL measurements were conducted by a confocal μ‐PL system (WITec, alpha‐300) using a 325 nm He Cd continuous wave laser and a 405 nm laser diode as the excitation sources. The size of the light spot focused on the samples was ≈0.1 mm. The output power could be adjusted from 30 to 0.3 mW using a neutral optical attenuator. The luminescence was dispersed by a triple‐grating 50 cm monochromator and detected by a GaAs photomultiplier tube using the conventional lock‐in technique. UPS measurements were performed on a PHI VersaProbe III instrument with He I 21.2 eV at 80.0 W, and the beam diameter was 5 µm. GIWAXS were performed on a Xeuss 2.0 SAXS/WAXS system (Xenocs SA, France). Cu Kα X‐ray source (GeniX3D Cu ULD), generated at 50 kV and 0.6 mA, was utilized to produce X‐ray radiation with a wavelength of 1.5418 Å. A semiconductor detector (Pilatus 300 K, DECTRIS, Swiss) with a resolution of 487×619 pixels (pixel size  =  172×172 µm^2^) was used to collect the scattering signals.

### Fabrication and Performance Measurement of FG‐PT Devices and Array

We first fabricated metal gate electrodes (Ti/Au: 5/50 nm) on p‐doped silicon wafer with 300 nm SiO_2_ by standard photolithography (ABM/6/350/NUV/DCCD/M), electron‐beam evaporation (ULVAC ei‐501z) and lift‐off processes (Remover and acetone). Subsequently, a 5 nm HfO_2_ tunneling layer was deposited via atomic layer deposition (ALD) using an ULTRATECH Savannah S100 system. The deposition was carried out at 200 °C under a N_2_ carrier gas flow of 20 sccm. The hafnium precursor (tetrakis (dimethylamido) hafnium (Hf (NMe_2_)_4_)) and the oxygen precursor (H_2_O) were introduced into the reaction chamber in pulsed sequences, synchronized with the carrier gas flow. Afterward, the ZnO QDs@CsPbBr_3_ solution was spin coated with a speed of 3000 rpm for 60 s. Subsequently, a layer of 5 nm thick HfO_2_ by atomic layer deposition was deposited and patterned into the desired pattern (40 µm×120 µm) using standard photolithography and reactive ion etching (Samco/RIE‐10NR) under CF_4_ gas at a flow rate of 50 sccm, a chamber pressure of 5 Pa, and a power setting of 100 W. Excess ZnO QDs@CsPbBr_3_ was removed by washing with a 0.01 mol L^−1^ HCl solution. Then, 30 nm thick HfO_2_ dielectric layer was deposited by atomic layer deposition. After that, the substrate was coated with a monolayer of hexamethyldisilazane and immersed in the semiconducting CNT solution at 60 °C for 2 h, followed by washing in toluene and isopropyl alcohol for 5 min each.^[^ ^38^ ^]^ Subsequently, the CNT film was patterned using photolithography followed by oxygen plasma etching (Yamato PR500) under the following conditions: an O_2_ flow rate of 180 sccm, a power of 200 W, and a duration of 2 min. This process formed channels with dimensions of 30 µm×100 µm. Finally, source and drain electrodes (Ti/Au: 5/50 nm) were fabricated by standard photolithography, electron‐beam evaporation, and lift‐off processes. The preparation procedure of FG‐PT solely using ZnO QDs and CsPbBr_3_ as FG was the same as described above.

The array was prepared using a different channel size (15 µm×50 µm). The simultaneous reduction in channel length and width allows the device to enhance integration density without significantly altering its performance. In addition, an additional layer of HfO_2_ (15 nm) was introduced to prevent crosstalk between devices. This dielectric layer avoids direct connection between the ground line and the word line, which guarantees each FG‐PT device operates independently.

The electrical measurements of FG‐PT were performed under ambient conditions with an Agilent‐B1500A semiconductor parameter analyzer and a Cascade M150 probe station. For the transfer curve measurements, the drain electrode was kept grounded, the *V*_DS_ = 1 V was applied on the drain electrode, and the gate voltage varied from −5 to 5 V. For photo response measurements of FG‐PT devices, first, a *V*_GS_ of 5 V was applied to transition to HRS, then light pulses of 516 nm (405 nm) of 5 ms (10 ms), 10 ms (20 ms), and 20 ms (30 ms) with intensity of 15 µW cm^−2^ (10 µW cm^−2^), 21 µW cm^−2^ (30 µW cm^−2^), and 42 µW cm^−2^ (39 µW cm^−2^) were applied, which were controlled by a laser controller (Thorlabs, ITC4001). Optical images were collected by a Nikon LV‐ND100 microscope. Electrical pulses of 5 or −5 V with a pulse width of 1 ms were applied to the gate, obtaining the current variation curve under the electrical pulses.

### DFT Calculations

First‐principles calculations are performed within the framework of density functional theory (DFT) as implemented in the Vienna ab initio simulation package (VASP), by using the projector augmented wave (PAW) pseudopotential. The PAW potential describes the [Ar] states of Zn, 1s^2^ states of O, [Kr]4d^10^ states of Cs, [Xe]4f^14^ states of Pb and [Ar]3d^10^ states of Br as core states. The exchange‐correlation functional is within the generalized gradient approximation (GGA), forward by Perdew, Burke, and Ernzerhof (PBE). The plane wave cutoff energy is set to 520 eV, and the Brillouin zone was sampled by a Γ‐centered 4×4×1 k‐grid mesh. The convergence criteria for the total energy and ionic forces are set to 10^−6^ and 0.01 eV Å^−1^, respectively. The spin‐orbit coupling (SOC) is included, allowing for the calculation of the electronic structure.

### Image Preprocessing of Cat Face and Scenery Images and Simulation of ANN Network for Cat Face Recognition

The output was the input image through different convolution kernels, where the CMOS convolution kernel is fully standardized with numerical values based on Python, and the convolution kernel used by the FG‐PT device normalizes the 100–200th impedance state to a number between −2 and 2 as the value of the convolution kernel. The CMOS convolution kernel use a precision of Python's default 64‐bit floating‐point precision, which does not require additional normalization. Horizontal convolution kernels extract edge information in the horizontal direction, while vertical convolution kernels extract edge information in the vertical direction. The complete image feature map was obtained by adding the images containing the two edges information. In addition to the cat face mentioned in the article, the feature map of the scenery was also obtained through the above method. Feature maps for the CMOS convolution kernel and the FG‐PT convolution kernel are input together with the original images into an ANN network. The original dataset, consisting of 11,144 images, was divided into a training set (80%) and an independent test set (20%) using random stratified sampling. The composition of the ANN network is to transform the input image into 4096 feature vectors, apply the ReLU function to the feature vectors, and then connect them to 1000 feature values. Finally, these 1000 feature values are divided into 190 categories for 190 different cats. During the training process, 64 data points were fed per epoch. Additionally, four key metrics from confusion matrix analysis were used: accuracy, precision, recall, and F1 score.

## Conflict of Interest

The authors declare no conflict of interest.

## Author Contributions

J.X. and B.T. contributed equally to this work. J.D. and L.M. conceived and supervised the project; J.X. and B.T. performed the experiments with the help of N.D., T.L., D.Z., Y.L. and S.Q; Z.L. characterized the crystal structure of ZnO QDs@CsPbBr_3_ PHC; J.X., B.T., J.D. analyzed the data and wrote the manuscript; All the authors discussed the results and commented on the manuscript.

## Supporting information



Supporting Information

## Data Availability

The data that support the findings of this study are available from the corresponding author upon reasonable request.

## References

[1] S. Deng , H. Zhao , W. Fang , J. Yin , S. Dustdar , A. Zomaya , IEEE Internet Things J. 2020, 7, 7457.

[2] P. McEnroe , S. Wang , M. Liyanage , IEEE Internet Things J. 2022, 9, 15435.

[3] K. Chen , H. Hu , I. Song , H. Gobeze , W. Lee , A. Abtahi , K. Schanze , J. Mei , Nat. Photonics 2023, 17, 629.

[4] B. Tong , J. Xu , J. Du , P. Liu , T. Du , Q. Wang , L. Li , Y. Wei , J. Li , J. Liang , C. Liu , Z. Liu , C. Li , L. Ma , Y. Chai , W. Ren , Nat. Commun. 2025, 16, 1609. 39948074 10.1038/s41467-025-56819-5 PMC11825893

[5] K. Liu , T. Zhang , B. Dang , L. Bao , L. Xu , C. Cheng , Z. Yang , R. Huang , Y. Yang , Nat. Electron. 2022, 5, 761.

[6] S. Lee , R. Peng , C. Wu , M. Li , Nat. Commun. 2022, 13, 1485. 35304489 10.1038/s41467-022-29171-1 PMC8933397

[7] Z. Zhang , S. Wang , C. Liu , R. Xie , W. Hu , P. Zhou , Nat. Nanotechnol. 2021, 17, 27. 34750561 10.1038/s41565-021-01003-1

[8] W. Li , T. Mu , Y. Chen , M. Dai , P. Sun , J. Li , W. Li , Z. Chen , Z. Wang , R. Yang , Z. Chen , Y. Wang , Y. Wu , S. Wang , Micro Nanostruct. 2024, 187, 207764.

[9] T. Wang , J. Meng , Z. He , L. Chen , H. Zhu , Q. Sun , S. Ding , P. Zhou , D. Zhang , Adv. Sci. 2020, 7, 1903480. 10.1002/advs.201903480 PMC7175259 32328430

[10] R. Jin , J. Wang , K. Shi , B. Qiu , L. Ma , S. Huang , Z. Li , RSC Adv. 2020, 10, 43225. 35514915 10.1039/d0ra08021g PMC9058139

[11] H. Lai , Y. Zhou , H. Zhou , N. Zhang , X. Ding , P. Liu , X. Wang , W. Xie , Adv. Mater. 2022, 34, 2110278. 10.1002/adma.202110278 35289451

[12] J. Pei , X. Wu , W. Liu , D. Zhang , S. Ding , ACS Nano 2022, 16, 2442. 35088590 10.1021/acsnano.1c08945

[13] Q. Li , T. Li , Y. Zhang , H. Zhao , J. Li , J. Yao , Nanoscale 2021, 13, 3295. 33533792 10.1039/d0nr09066b

[14] D. Christensen , R. Dittmann , B. Linares‐Barranco , A. Sebastian , M. Gallo , A. Redaelli , S. Slesazeck , T. Mikolajick , S. Spiga , S. Menzel , I. Valov , G. Milano , C. Ricciardi , S. Liang , M. Lanza , T. Quill , S. Keene , A. Salleo , J. Grollier , D. Marković , A. Mizrahi , P. Yao , J. Yang , G. Indiveri , J. Strachan , S. Datta , E. Vianello , A. Valentian , J. Feldmann , X. Li , et al., Neuromorph. Comput. Eng. 2022, 2, 022501.

[15] A. Mehonic , A. Kenyon , Nature 2022, 604, 255. 35418630 10.1038/s41586-021-04362-w

[16] H. Ning , Z. Yu , Q. Zhang , H. Wen , B. Gao , Y. Mao , Y. Li , Y. Zhou , Y. Zhou , J. Chen , L. Liu , W. Wang , T. Li , Y. Li , W. Meng , W. Li , Y. Li , H. Qiu , Y. Shi , Y. Chai , H. Wu , X. Wang , Nat. Nanotechnol. 2023, 18, 493. 36941361 10.1038/s41565-023-01343-0

[17] Y. Hou , Y. Li , Z. Zhang , J. Li , D. Qi , X. Chen , J. Wang , B. Yao , M. Yu , T. Lu , J. Zhang , ACS Nano 2021, 15, 1497. 33372769 10.1021/acsnano.0c08921

[18] G. Li , D. Xie , H. Zhong , Z. Zhang , X. Fu , Q. Zhou , Q. Li , H. Ni , J. Wang , E.‐j. Guo , M. He , C. Wang , G. Yang , K. Jin , C. Ge , Nat. Commun. 2022, 13, 1729. 35365642 10.1038/s41467-022-29456-5 PMC8975822

[19] Z. Luo , X. Xia , M. Yang , N. Wilson , A. Gruverman , M. Alexe , ACS Nano 2020, 14, 746. 31887010 10.1021/acsnano.9b07687

[20] Q. Chen , H. Zhou , Z. Hong , S. Luo , H. S. Duan , H. H. Wang , Y. Liu , G. Li , Y. Yang , J. Am. Chem. Soc. 2014, 136, 622. 24359486 10.1021/ja411509g

[21] H. Chu , R. Li , P. Feng , D. Wang , C. Li , Y. Yu , M. Yang , ACS Catal. 2024, 14, 1553.

[22] Z. Liu , J. Hu , H. Jiao , L. Li , G. Zheng , Y. Chen , Y. Huang , Q. Zhang , C. Shen , Q. Chen , H. Zhou , Adv. Mater. 2017, 29, 1606774. 10.1002/adma.201606774 28417481

[23] Y. Zhang , Y. Wang , L. Zhao , X. Yang , C. H. Hou , J. Wu , R. Su , S. Jia , J. J. Shyue , D. Luo , P. Chen , M. Yu , Q. Li , L. Li , Q. Gong , R. Zhu , Energy Environ. Sci. 2021, 14, 6526.

[24] Y. Ba , W. Zhu , Z. Xu , S. Jiang , M. Yang , F. Bai , H. Xi , D. Chen , J. Zhang , C. Zhang , Y. Hao , ACS Appl. Mater. Interfaces 2024, 16, 55783. 39361505 10.1021/acsami.4c12010

[25] L. Li , Y. Li , J. Li , Y. Fang , D. Yang , Adv. Opt. Mater. 2023, 11, 2202276.

[26] L. Li , H. Yan , S. Li , H. Xu , D. Qu , A. Hu , L. Ma , Y. Ji , Q. Zhong , L. Zhao , F. Xu , Y. Tu , T. Song , J. Wu , M. Li , C. Lu , X. Yang , H. Zhong , Q. Gong , X. Wang , R. Zhu , Adv. Mater. 2024, 36, 2409201. 10.1002/adma.202409201 39498664

[27] Y. Zhou , L. Zhao , Z. Ni , S. Xu , J. Zhao , X. Xiao , J. Huang , Sci. Adv. 2021, 7, abg6716. 10.1126/sciadv.abg6716 PMC8442865 34516903

[28] Y. Jiang , C. Sun , J. Xu , S. Li , M. Cui , X. Fu , Y. Liu , Y. Liu , H. Wan , K. Wei , T. Zhou , W. Zhang , Y. Yang , J. Yang , C. Qin , S. Gao , J. Pan , Y. Liu , S. Hoogland , E. H. Sargent , J. Chen , M. Yuan , Nature 2022, 612, 679. 36543955 10.1038/s41586-022-05486-3

[29] P. Zhang , Y. Hua , Y. Xu , Q. Sun , X. Li , F. Cui , L. Liu , Y. Bi , G. Zhang , X. Tao , Adv. Mater. 2022, 34, 2106562. 10.1002/adma.202106562 35062044

[30] J. Duan , Y. Zhao , B. He , Q. Tang , Angew. Chem. 2018, 57, 3787. 29380514 10.1002/anie.201800019

[31] M. Shoaib , X. Zhang , X. Wang , H. Zhou , T. Xu , X. Wang , X. Hu , H. Liu , X. Fan , W. Zheng , T. Yang , S. Yang , Q. Zhang , X. Zhu , L. Sun , A. Pan , J. Am. Chem. Soc. 2017, 139, 15592. 29058888 10.1021/jacs.7b08818

[32] T. Xuan , X. Yang , S. Lou , J. Huang , Y. Liu , J. Yu , H. Li , K. Wong , C. Wang , J. Wang , Nanoscale 2017, 9, 15286. 28975949 10.1039/c7nr04179a

[33] G. Zhang , S. Hou , H. Zhang , W. Zeng , F. Yan , C. Li , H. Duan , Adv. Mater. 2015, 27, 2400. 25728828 10.1002/adma.201405222

[34] J. Li , L. Liu , Q. Liang , M. Zhou , C. Yao , S. Xu , Z. Li , J. Hazard. Mater. 2021, 414, 125395. 33652218 10.1016/j.jhazmat.2021.125395

[35] K. Zheng , A. Boccaccini , Adv. Colloid Interface Sci. 2017, 249, 363. 28364954 10.1016/j.cis.2017.03.008

[36] J. Endres , D. Egger , M. Kulbak , R. Kerner , L. Zhao , S. Silver , G. Hodes , B. Rand , D. Cahen , L. Kronik , A. Kahn , J. Phys. Chem. Lett. 2016, 7, 2722. 27364125 10.1021/acs.jpclett.6b00946 PMC4959026

[37] Y. Hinuma , A. Grueneis , G. Kresse , F. Oba , Phys. Rev. B 2014, 90, 155405. 10.1103/PhysRevLett.112.096401 24655265

[38] Q. Zhu , B. Li , D. Yang , C. Liu , S. Feng , M. Chen , Y. Sun , Y. Tian , X. Su , X. Wang , S. Qiu , Q. Li , X. Li , H. Zeng , H. Cheng , D. Sun , Nat Commun. 2021, 12, 1798. 33741964 10.1038/s41467-021-22047-w PMC7979753

