# Supplementary material for: ZnO Quantum Dots@CsPbBr3 Poly‐Heterocrystalline Film Enables High‐Performance Floating‐Gate Transistor Arrays for Edge Computing
Source: Adv Sci (Weinh). 2025 Aug 30;12(43):e06357. doi: 10.1002/advs.202506357 (PMC12631869; doi:10.1002/advs.202506357)
Supplement: Supplementary file 1 — Supporting Information [file ADVS-12-e06357-s001.docx]

Supporting Information for

**ZnO quantum dots@CsPbBr_3_ poly-heterocrystalline film enables high-performance floating-gate transistor arrays for edge computing**

Jiajun Xu,^1,2†^ Bo Tong,^1,2†^ Nian Dai,^1,2^ Tongjian Liu,^1^ Zhibo Liu,^1,2^ Dingdong Zhang,^1,2^ Yan Liang,^1^ Song Qiu,^3^ Lai-Peng Ma,^1,2^ Jinhong Du^1,2*^

^1^Shenyang National Laboratory for Materials Science, Institute of Metal Research, Chinese Academy of Sciences, Shenyang 110016, China

^2^School of Materials Science and Engineering, University of Science and Technology of China, Shenyang 110016, China

^3^Advanced Materials Division, Suzhou Institute of Nano-Tech and Nano-Bionics, Chinese Academy of Sciences, Suzhou 215123, China

*Corresponding author. Email: jhdu@imr.ac.cn

^†^These authors contributed equally.

**The PDF file includes:**

Notes S1 to S5

Figures S1 to S30

Tables S1 to S3

References

**Notes S1 to S5**

**Note S1.** Calculation of exciton lifetime of CsPbBr_3_ and ZnO QDs@CsPbBr_3_ PHC

Time-Resolved Photoluminescence (TRPL) decay curves were probed at 532 nm wavelengths with different mass fractions of ZnO QDs from 0% to 2.4% (Figure 2g). We fitted the curves by using the following formula to obtain exciton lifetime *τ*_0_. It is found that *τ*_0_ is increased from 1.77 ns for CsPbBr_3_ to 3.18 ns for ZnO QDs@CsPbBr_3_ PHC with mass fraction of ZnO QDs of 2.4%.

$$\text{I}\text{(t)}\text{ }\text{= }I_{0}\text{e}^{-t/\text{τ}_{\text{0}}}$$

Where *I*(t), *I*_0_, and *t* are light intensity, initial light intensity and time, respectively.

**Note S2.** Calculation of electrical power consumption of FG-PT

We have used the following formula to calculate the electrical energy consumption of FG-PT.

$$E=V\times I\times\Delta t$$

Where *V*, $I$ and $\Delta t$ mean gate-source voltage, gate-source current, and voltage pulse width, respectively.

To generate a non-volatile state, *V*_GS_ is 1 V, *I*_GS_ is 178 fA (Figure S15) and pulse width is 1 s, which results in an ultralow power consumption of about 178 fJ.

**Note S3.** The charge storage density of the floating gate layer

The charge storage density of the FG was calculated using classical method^[1,2]^. The calculation formula for charge storage density (*n*) is as follows:

$$n= \frac{{\Delta V}_{th}\cdot C_{FG}}{A\cdot q}$$

Where Δ*V*_th_ is the threshold voltage shift; *q* is the elementary charge of 1.6 × 10^-19^ C; *A* is floating gate area; *C*_FG_ refers to dielectric capacitance of float gate, which can be determined by the following formula:

$$C_{FG}=\varepsilon_{0}\cdot\varepsilon_{r}\cdot A/d$$

Where *ε*_₀_ is the vacuum permittivity of 8.85 × 10^-12^ F/m, *ε*_r_ represents relative permittivity, and *d* is the thickness of dielectric layer.

For our device, the transfer characteristics curve (Figure 3b) shows a Δ*V*_th_ of 3.2 V. The dielectric layer is HfO_2_ with a *ε*_r_ of 20, a thickness *d* of 30 nm, and a floating gate area *A* of 3000 μm^2^ (30 μm×100 μm) (Figure S10). The calculation yields n ≈ 1.18×10^13^ cm^-2^, which exceeds the charge storage density of the references^[1,2]^. This is mainly attributed to two aspects. First, ZnO QDs@CsPbBr_3_ has excellent conductivity, enabling the FG-PT device to store more charge per unit area. Second, the band structure analysis reveals that the valence band maximum of ZnO QDs@CsPbBr_3_ presents an energy barrier of ~1.6 eV (Figure S16b) in combined with the tunneling layer (HfO_2_), effectively preventing hole leakage.

**Note S4.** The role of 15 nm HfO_2_ layer in FG-PT array

The 15 nm HfO_2_ layer prevents direct contact between the ‘word line’ and ‘ground line’ (Figure S26), thereby avoiding short-circuit issues. We measured the leakage currents between adjacent word lines (word line1-word line2) and adjacent ground lines (ground line1-ground line2) under a voltage of 1 V. The leakage current was at the femtoampere (fA) level, confirming the effectiveness of 15 nm HfO_2_ to avoid short circuits (Figures S27a and b). Moreover, 15 nm HfO_2_ layer effectively suppresses the crosstalk in FG-PT array during hole writing and erasing processes. When *V*_GS_ (5 V) is applied to the (1,1) device (row 1, column 1) via the 'bit line' to switch it from the LRS to the HRS, we simultaneously measured the leakage current between ground line 1 and ground line 2 of the adjacent (1,2) device (Figure S27c). Similarly, during hole erasing process, applying a light pulse to the (1,1) device, we simultaneously measured the leakage current between ground line 1 and ground line 2 of (1,2) device (Figure S27d), and between word line 1 and word line 2 of (2,1) device (Figure S27e), respectively. The results show the leakage currents (<10^-13^A) are several orders of magnitude lower than the operational *I*_DS_ (>10^-7^ A) of the FG-PT device (Figure 3b-e), confirming negligible crosstalk and no significant device performance impact.

**Note S5.** The evaluation of ANN

The original dataset, consisting of 11,144 images, was divided into a training set (80%) and an independent test set (20%) using random stratified sampling. This partitioning ratio ensures sufficient training data while maintaining the statistical significance of the test set. Critically, the test set remained isolated throughout the model development and tuning process, and all reported performance indicators are derived from the evaluation results of this independent test set.

To comprehensively evaluate model performance, we used four key metrics from confusion matrix analysis: Accuracy, Precision, Recall, and F1 Score, which are obtained by the following equations.

$$Accuracy= \frac{TP+NP}{TP+NP+FP+FN}$$

$$Precision= \frac{TP}{TP+FP}$$

$$Recall= \frac{TP}{TP+FN}$$

$$F1 Score=2\cdot\frac{Precision\cdot Recall}{Precision+Recall}$$

True Positive (TP): The model correctly predicts an actual positive sample as positive.

False Negative (FN): The model incorrectly predicts an actual positive sample as negative.

False Positive (FP): The model incorrectly predicts an actual negative sample as positive.

True Negative (TN): The model correctly predicts an actual negative sample as negative.

Based on these evaluation metrics, we conducted a systematic comparative analysis of three schemes: Original, CMOS convolutional kernel, and FG-PT convolutional kernel (Figure S29). The experimental results show that while maintaining high accuracy, the FG-PT convolutional kernel significantly improves the model’s precision, recall and F1 Score. Since these four evaluation metrics respectively reflect the model's performance in different aspects (accuracy measures the overall correctness of predictions, precision focuses on the accuracy of predictions for positive samples, recall reflects the completeness of positive sample identification, and the F1 score comprehensively balances the first two), their simultaneous improvement indicates that the model has achieved a comprehensive and balanced optimization during the training process, rather than over-optimization for specific metrics or training data.

**Figures S1 to S30**

**
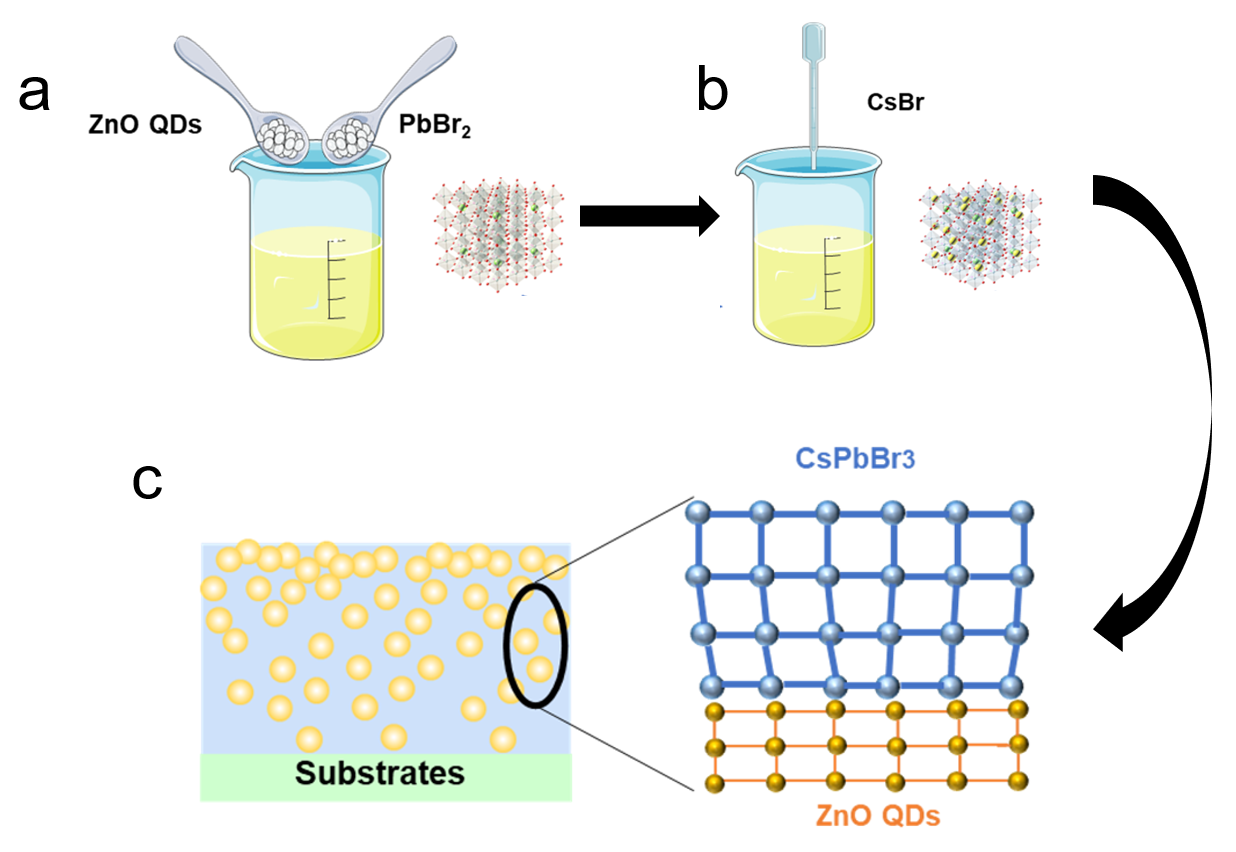
**

**Figure S1.** Schematic fabrication process of ZnO QDs@CsPbBr_3_ PHC film. a) PbBr_2_ was first mixed with ZnO QDs in DMSO solvent to form ZnO QDs@PbBr_2_ colloidal solution. b) CsBr was then added into the ZnO QDs@PbBr_2_ colloidal solution and heated at 60 ℃ for 6 h to form ZnO QDs@CsPbBr_3_ colloidal solution. c) ZnO QDs@CsPbBr_3_ PHC film was obtained through spin-coating followed by heating at 60 ℃ for 20 min.


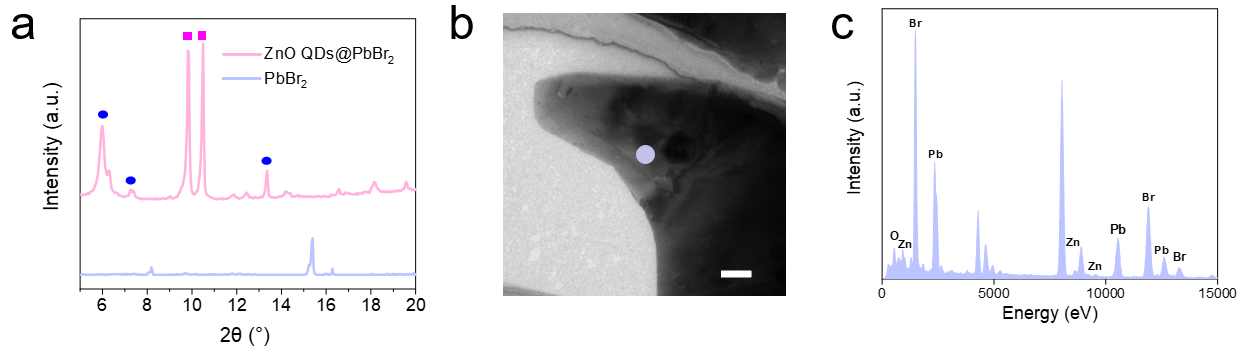


**Figure S2.** a) XRD patterns of PbBr_2_ and ZnO QDs@PbBr_2_ clusters, in which blue and pink points represent PbBr_2_ and ZnO QDs@PbBr_2_ characteristic peaks, respectively. b) The TEM image of ZnO QDs@CsPbBr_3_ PHC. Scale bar, 100 nm. c) The point TEM-EDS analysis of the ZnO QDs@CsPbBr_3_ PHC. Cs was not detected due to its low atomic weight. See Table S1 for details.


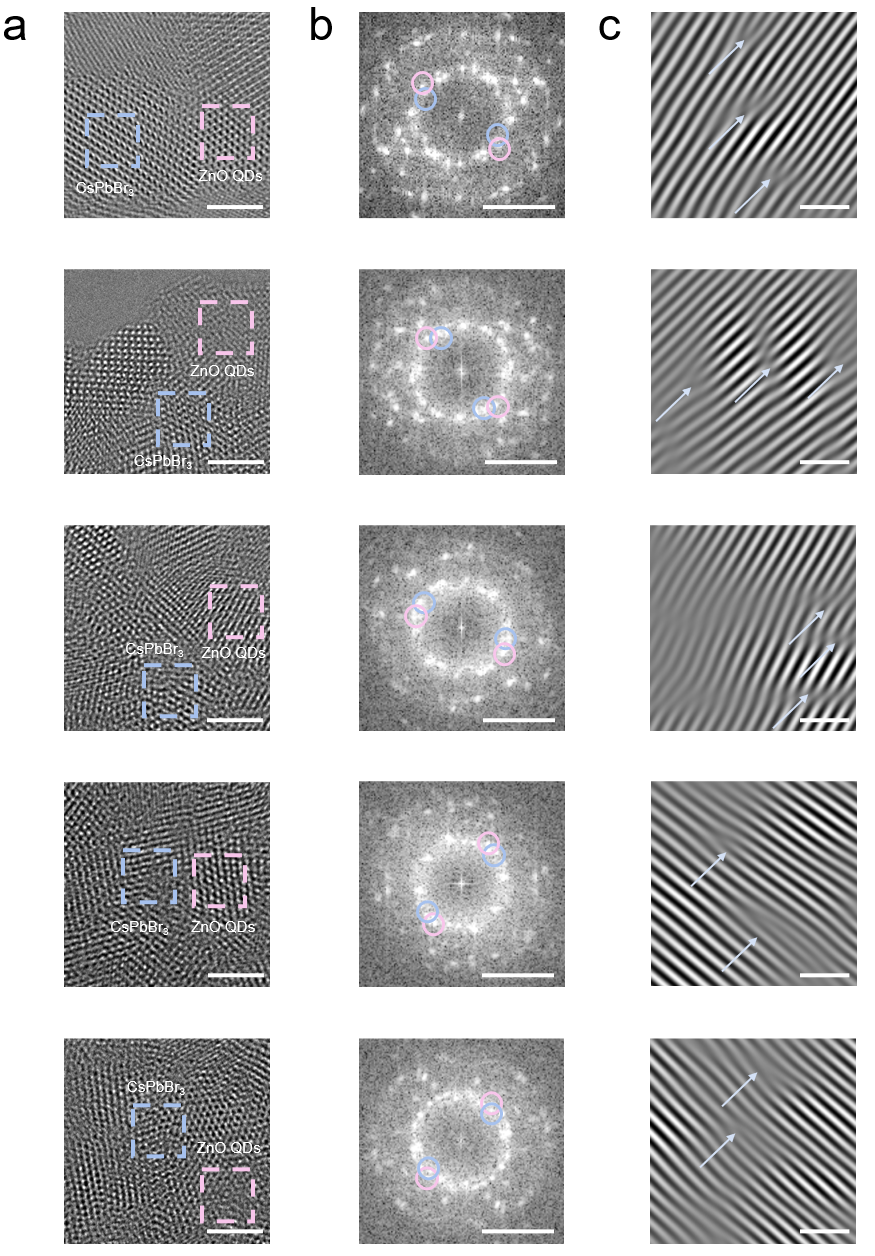


**Figure S3.** a) TEM images of ZnO QDs@CsPbBr_3_ PHC of five regions. Scale bars, 2 nm. b) The corresponding FFTs of ZnO QDs@CsPbBr_3_ PHC. The pink circles represent ZnO QDs, while the blue circles represent CsPbBr_3_. Scale bars, 6 nm^-1^. c) IFFT images of ZnO QDs@CsPbBr_3_ PHC. The arrows indicate the formation of a small number of dislocations at the interface between ZnO QDs and CsPbBr_3_. Scale bars, 0.5 nm.


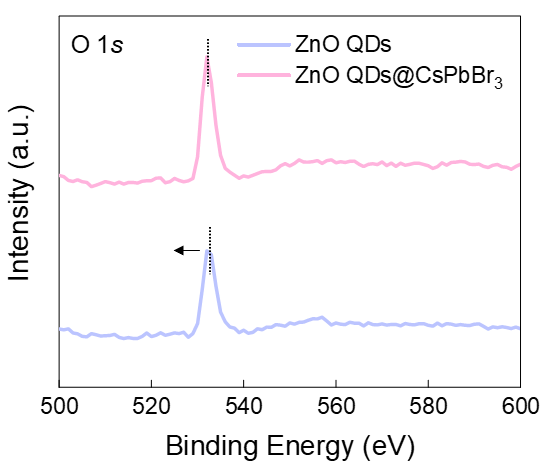


**Figure S4.** The O 1*s* XPS spectra of ZnO QDs and ZnO QDs@CsPbBr_3_.


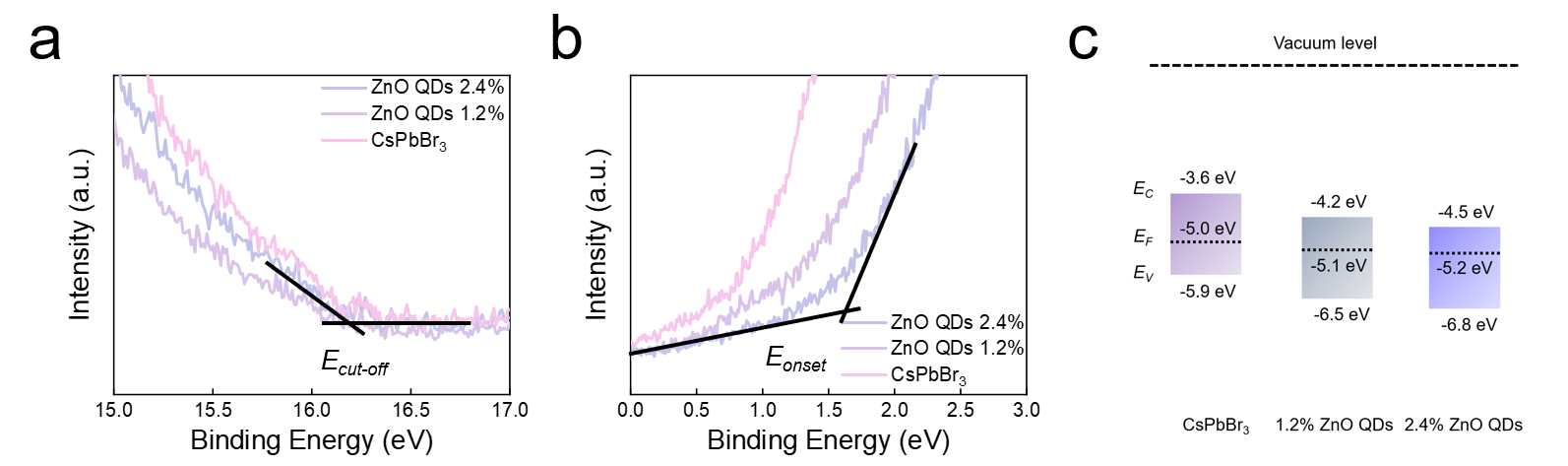


**Figure S5.** UPS spectra and band structure of CsPbBr_3_ and ZnO QDs@CsPbBr_3_ PHC film with different mass fractions of ZnO QDs. The low-energy cut-off edge (a) and the onset edge (b) of UPS spectra. The Fermi level (*E_F_*) and VBM (*E_V_*) of the materials can be calculated using the formulas: *E_F_*=-(*E_He I_*-*E_cut-off_*), *E*_V_=-(*E_onset_-E_F_*), where *E_He I_* is the energy of the He I laser used in UPS with a value of 21.2 eV, *E_cut-off_* and *E_onset_* corresponds to the cut-off edge and the onset edge. The *E_cut-off_* values for CsPbBr_3_, ZnO QDs@ CsPbBr_3_ PHC with 1.2%and 2.4% ZnO QDs are 16.2 eV, 16.1 eV, and 16.0 eV, respectively. Therefore, their *E*_F_ are -5.0 eV, -5.1 eV, and -5.2 eV, respectively. Their *E_onset_* values are 0.9 eV, 1.4 eV, and 1.6 eV, respectively, yielding *E*_V_ of -5.9 eV, -6.5 eV, and -6.8 eV, respectively. c) The band structure of CsPbBr_3_ and ZnO QDs@CsPbBr_3_ PHC with varying mass fractions of ZnO QDs. The PL spectra show that the peak position of CsPbBr_3_, and ZnO QDs@CsPbBr_3_ PHC with 1.2% ZnO QDs, 2.4% ZnO QDs is 524, 522 and 520 nm (Figure 2f) corresponding to the band gaps of 2.36 eV, 2.37 eV, and 2.38 eV, respectively. Thus, we obtained the CBM (*E_C_*) of -3.6, -4.2 and -4.5 eV for CsPbBr_3_, 1.2% and 2.4 ZnO QDs@CsPbBr_3_.


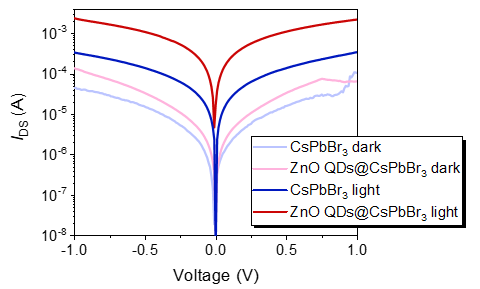


**Figure S6.** The output curves of CsPbBr_3_ and ZnO QDs@CsPbBr_3_ PHC film in the dark and under light. The light wavelength is 516 nm and light intensity is 1.95 mW/cm^2^. The mass fraction of ZnO QDs is 2.4%.


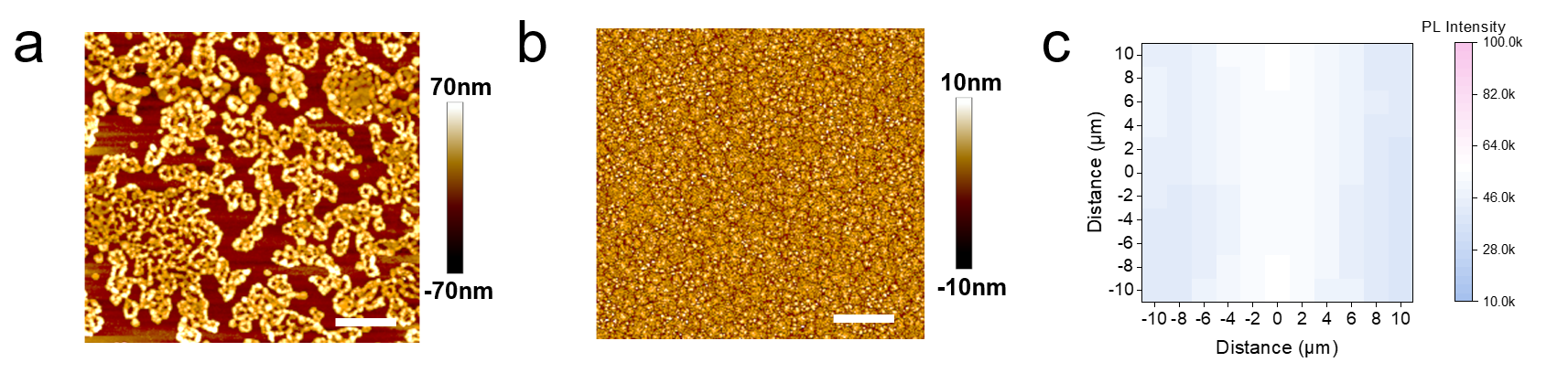


**Figure S7.** a) Atomic Force Microscopy (AFM) image of CsPbBr_3_ and b) ZnO QDs@CsPbBr_3_ PHC films. The mass fraction of ZnO QDs is 2.4% in ZnO QDs@CsPbBr_3_ PHC film. Scale bars, 1 μm. c) The PL mapping of ZnO QDs@CsPbBr_3_ PHC film.


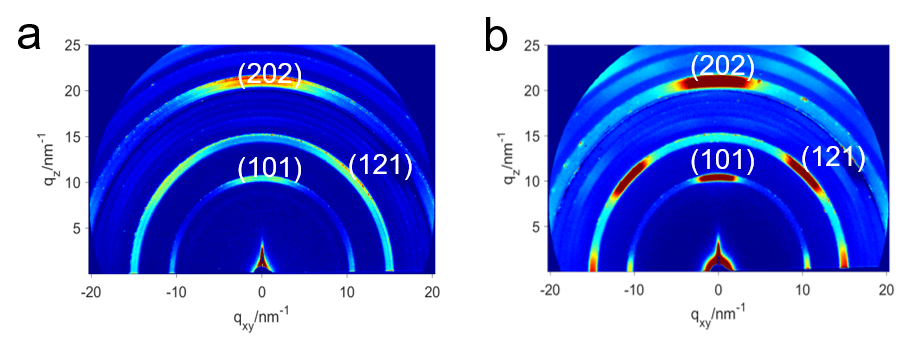


**Figure S8.** a) GIWAXS patterns of CsPbBr_3_ and b) ZnO QDs@CsPbBr_3_ PHC film. The mass fraction of ZnO QDs is 2.4% in ZnO QDs@CsPbBr_3_ PHC.


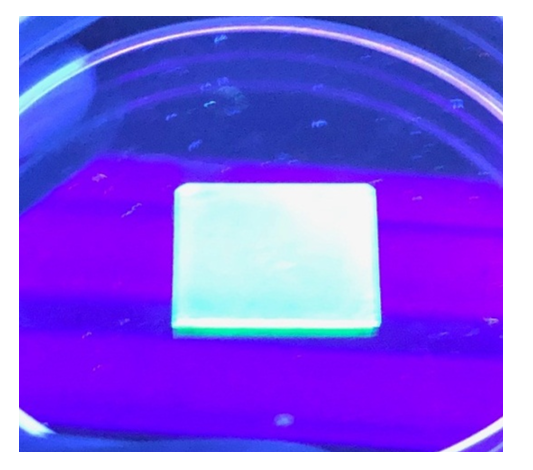


**Figure S9.** Photograph of ZnO QDs@CsPbBr_3_ PHC film under ultraviolet light after 1 month. The size of PHC film is 2 cm×2 cm.


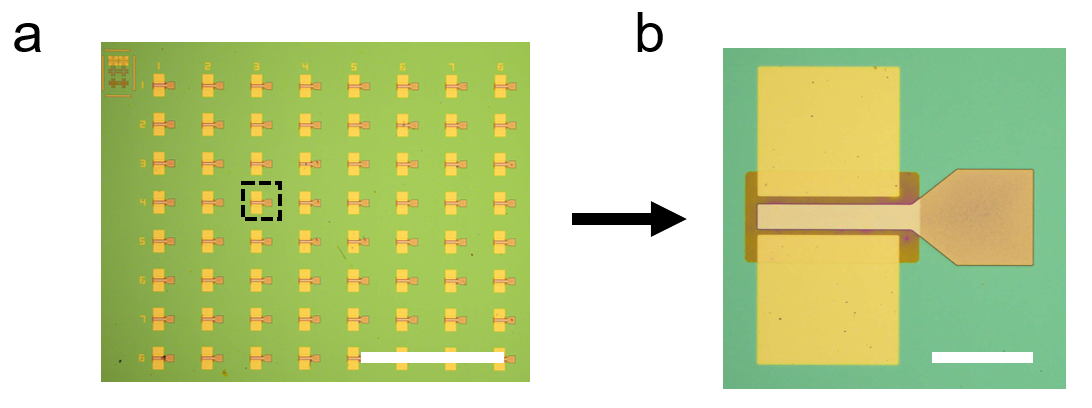


**Figure S10.** Optical microscopy images of the FG-PT devices. a) Overview of the device structure. Scale bar, 1 mm. b) Enlarged view of the box marked in (a). Scale bar, 100 μm.


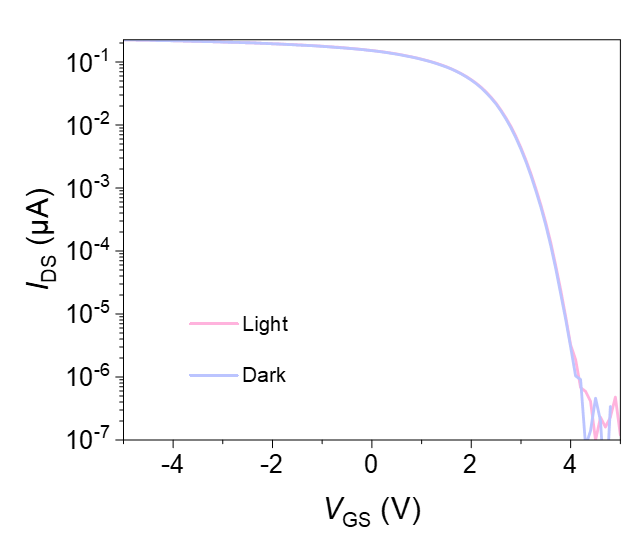


**Figure S11.** Transfer curves of CNT film in the dark and under light. The light intensity is 21 μW/cm^2^ and the wavelength is 516 nm. The similar curves imply CNTs have negligible photo-response.


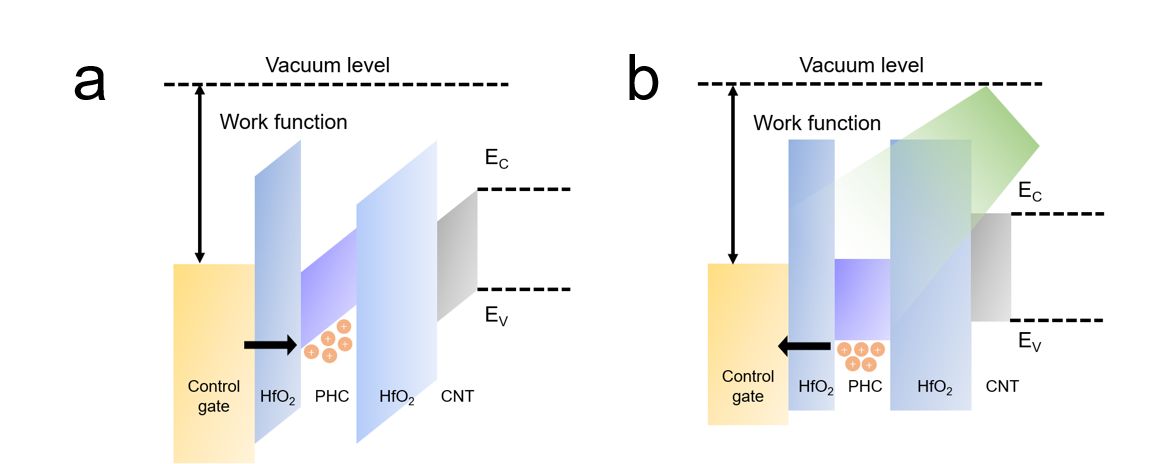


**Figure S12.** a) The band diagram of FG-PT device under positive gate voltage, holes will be written into the FG layer. The writing of holes causes FG-PT change from the initial state to high resistance state. b) The band diagram of FG-PT device under light pulse, holes in FG can be erased out of FG layer. The erasing of holes causes FG-PT change from high resistance state to initial state.


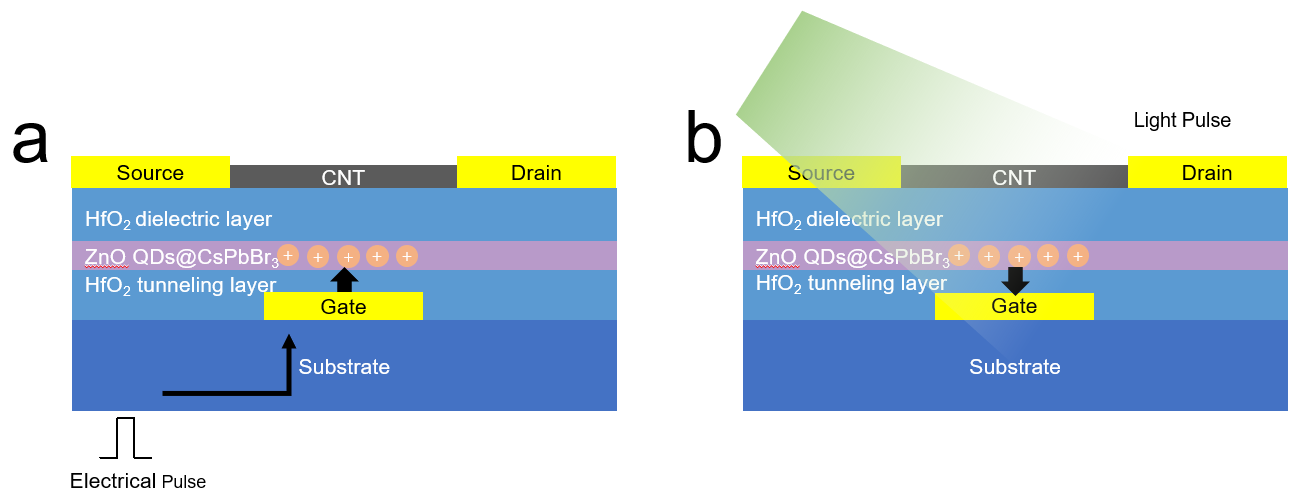


**Figure S13.** Schematic of holes writing into the FG layer or erasing out of the FG layer. a) Under positive voltage, holes will be written into the FG layer, and b) the holes can be erased out of the FG layer under light.


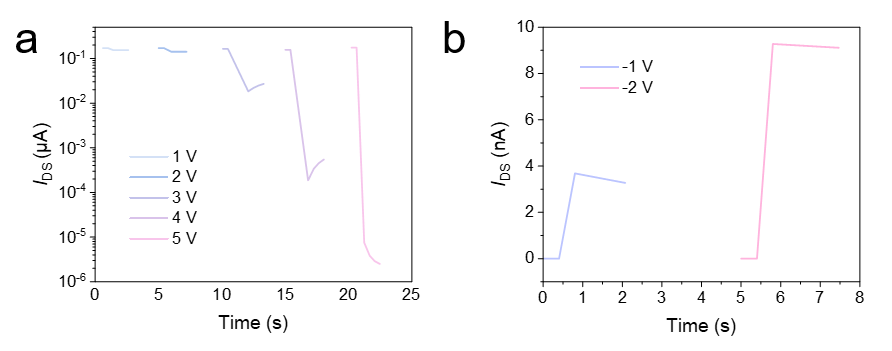


**Figure S14.** a) The electrical writing process of the FG-PT device under different positive *V*_GS_. The pulse widths are 400 ms. *V*_DS_=100 mV. b) The electrical erasing process of the FG-PT device under different genitive *V*_GS_. The pulse widths are 1 ms. *V*_DS_=100 mV.


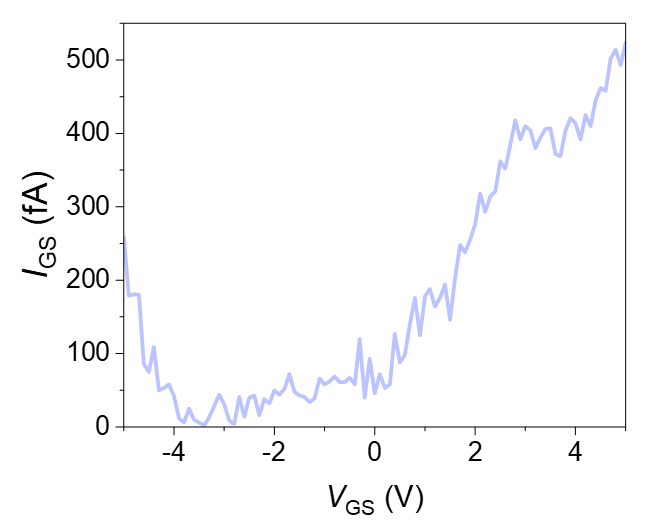


**Figure S15.** *I*_GS_- *V*_GS_ curve of FG-PT.


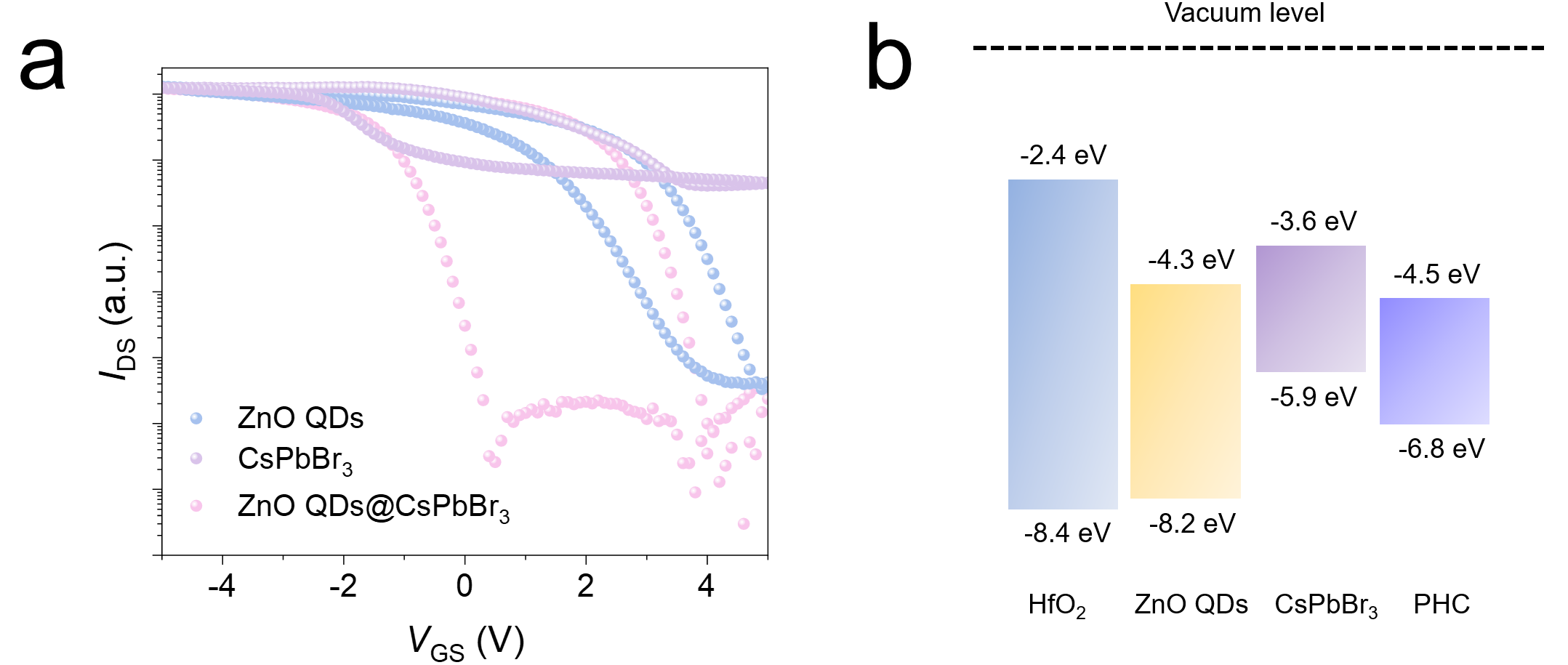


**Figure S16.** a) Transfer curves of the FG-PT device utilizing ZnO QDs, CsPbBr_3_ and ZnO QDs@ CsPbBr_3_ as floating-gate layer in dark. b) The band structure of HfO_2_^[3]^, ZnO QDs^[4]^, CsPbBr_3_ and ZnO QDs@CsPbBr_3_ PHC.


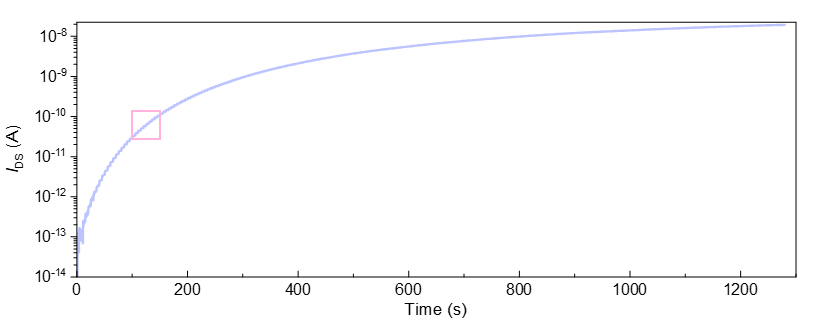


**Figure S17.** The light erasing process of FG-PT device, where the light pulse widths are 10 ms with 5 s intervals. The light wavelength is 516 nm with the light intensity of 21 μW/cm^2^. The image of Figure 3d is an enlarged view of the box.


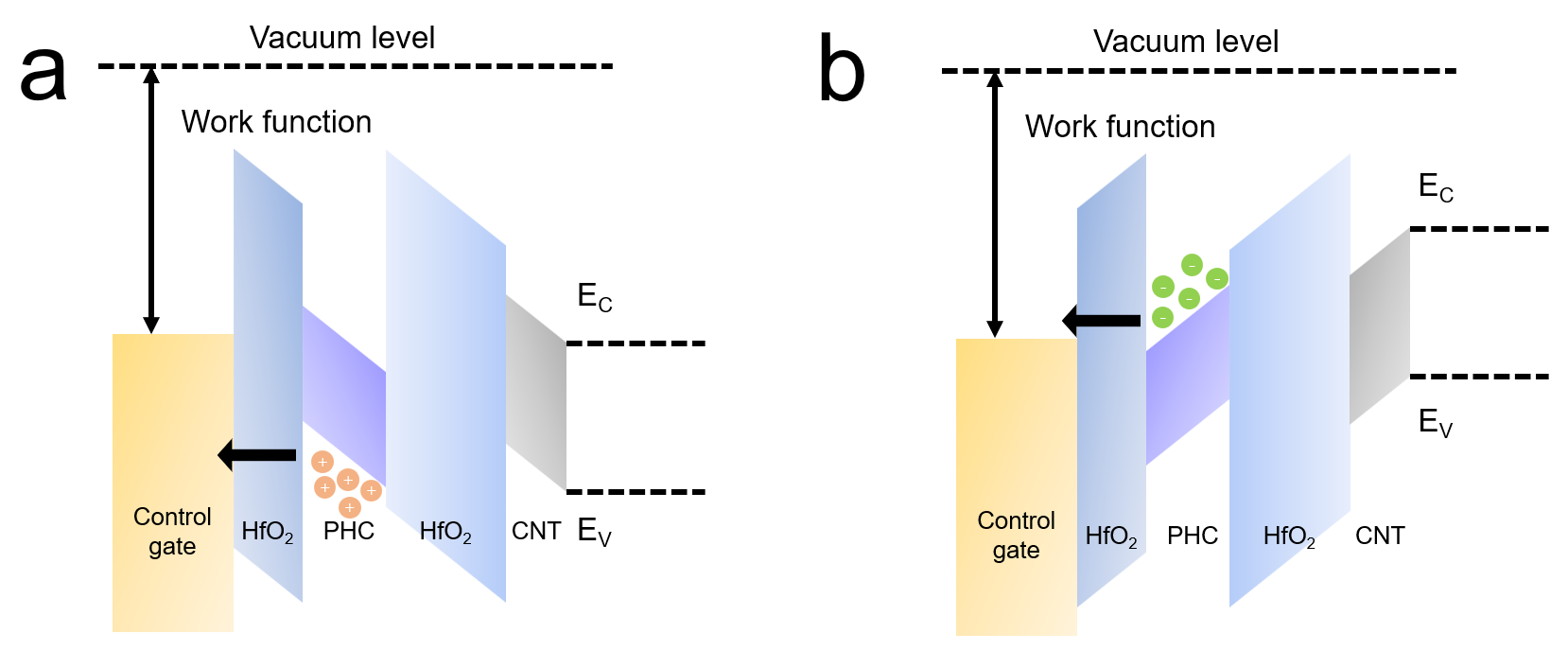


**Figure S18.** a) The band diagram of FG-PT device under negative gate voltage, holes can be erased out from the FG layer. b) The band diagram of FG-PT device under positive gate voltage, electrons can be erased out from the FG layer.


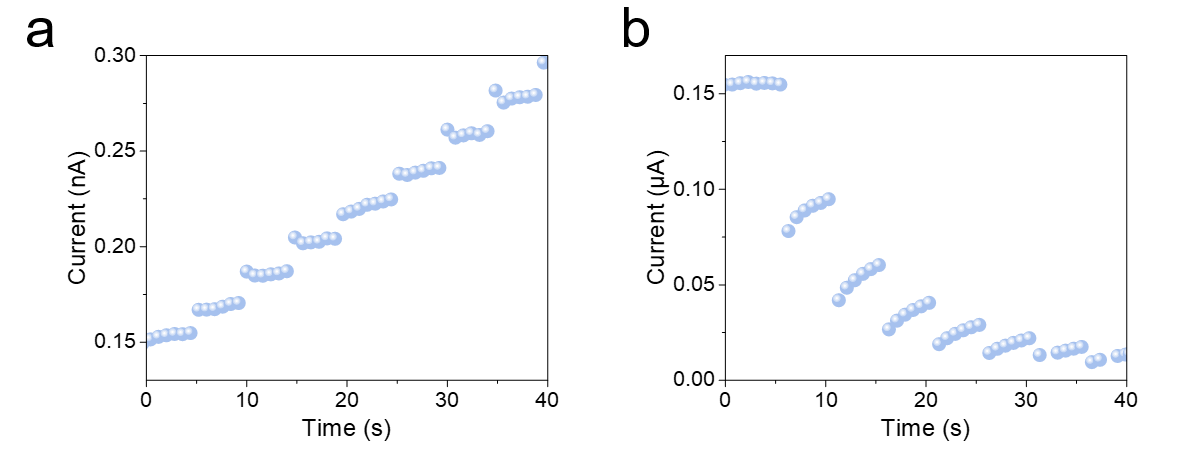


**Figure S19.** a) Long term potentiation behavior of FG-PT under electrical pulse. The applied *V*_GS_ is -5 V with the pulse width of 1 ms. b) Long term depression behavior of FG-PT under electrical pulse, the applied *V*_GS_ is 5 V with the pulse width of 1 ms.


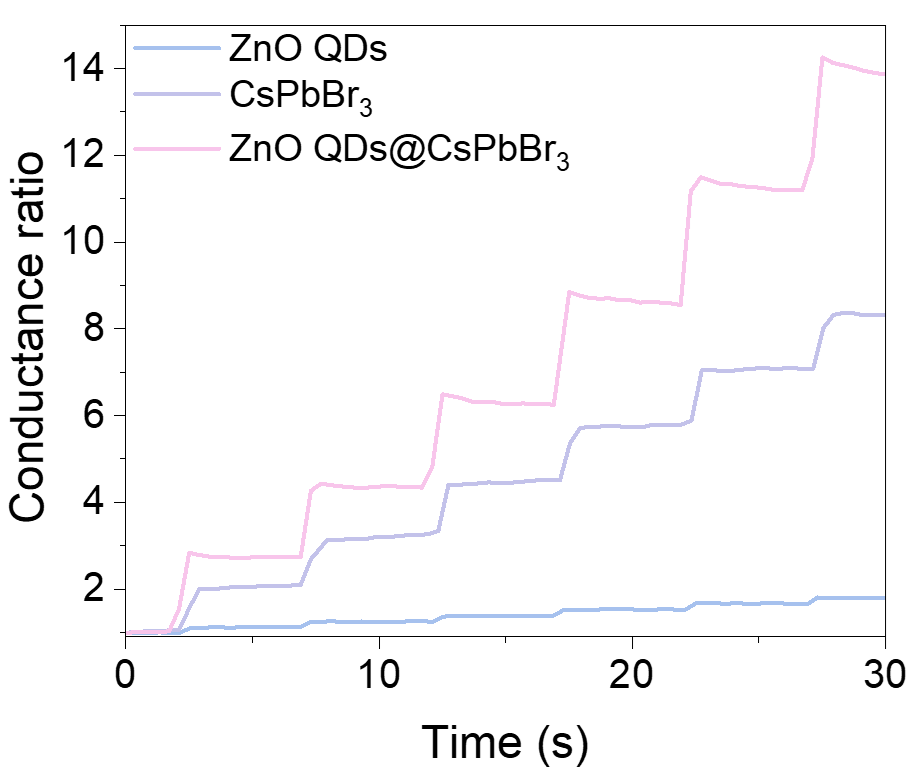


**Figure S20.** The light erasing process of FG-PT utilizing ZnO QDs, CsPbBr_3_ and ZnO QDs@ CsPbBr_3_ as floating gate layer. The light wavelength is 405 nm with light pulse width of 100 ms and the light intensity of 35 μW/cm^2^.


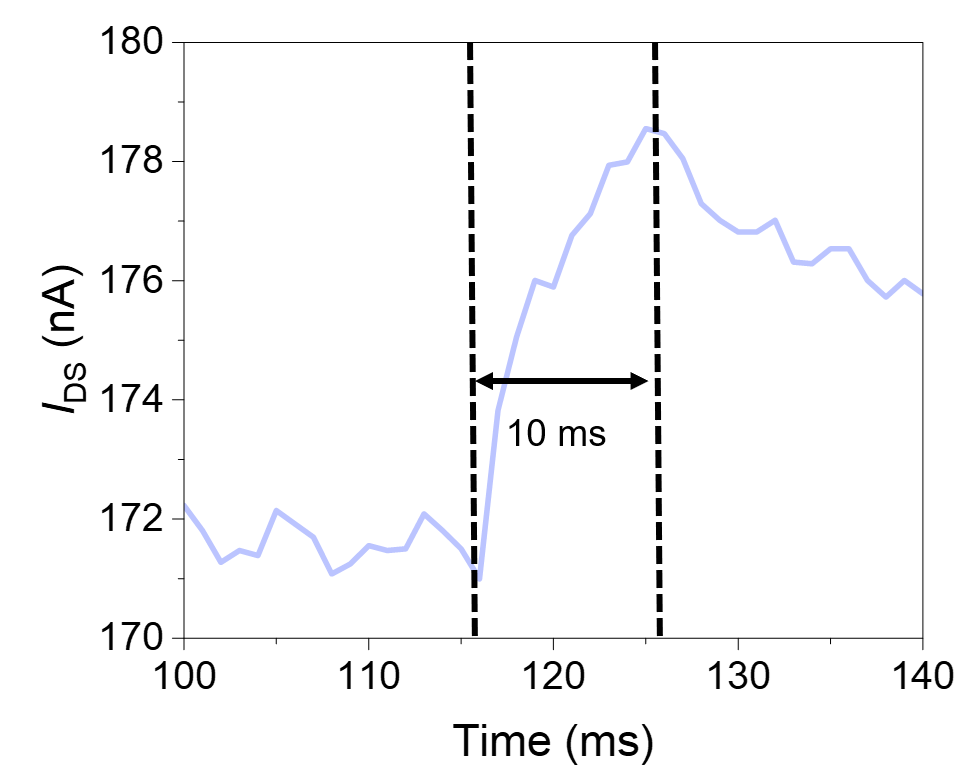


**Figure S21.** The photo-response of FG-PT under 405 nm light with pulse width of 10 ms. The light intensity is 20 μW/cm^2^. *V*_DS_=100 mV.


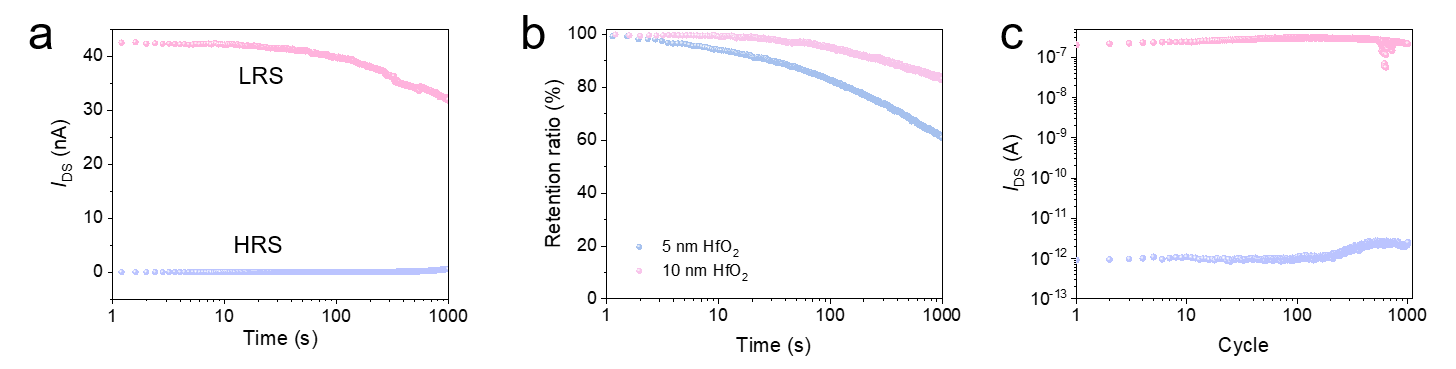


**Figure S22.** a) Retention of the FG-PT at HRS and LRS. b) Retention ratio of the FG-PT with 5 nm-thick and 10 nm-thick HfO_2_ tunneling layer at LRS. c) Cycle stability test of the FG-PT. We first apply a voltage pulse of 5 V for 1 s, and read the current of the HRS; then, we apply a voltage pulse of -5 V for 1 s to read the current of LRS.


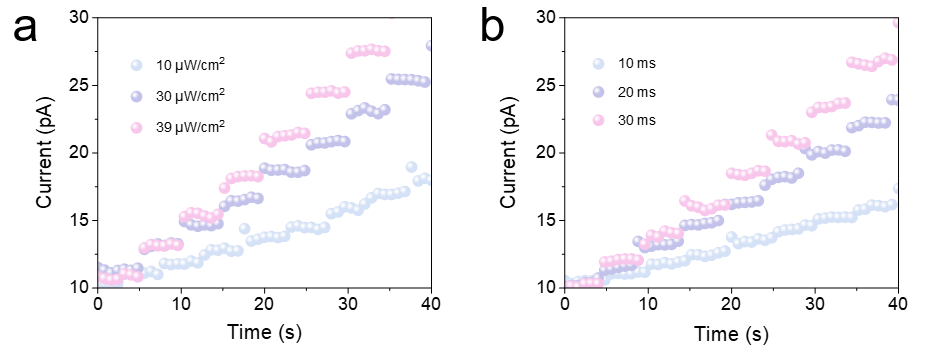


**Figure S23.** a) Modulation of synaptic weight of FG-PT under 405 nm laser with different light intensity. The light pulse width is 30 ms. b) Modulation of synaptic weight of FG-PT under 405 nm laser with different light pulse width. The light intensity is 30 μW/cm^2^.


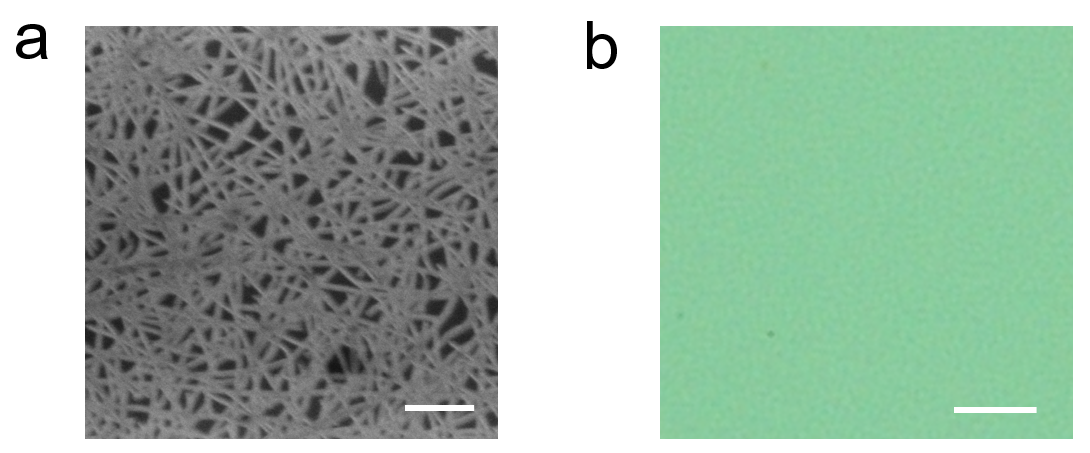


**Figure S24.** a) The SEM image of CNT film. Scale bar, 500 nm. b) The optical image of HfO_2_ film. Scale bar, 10 µm.


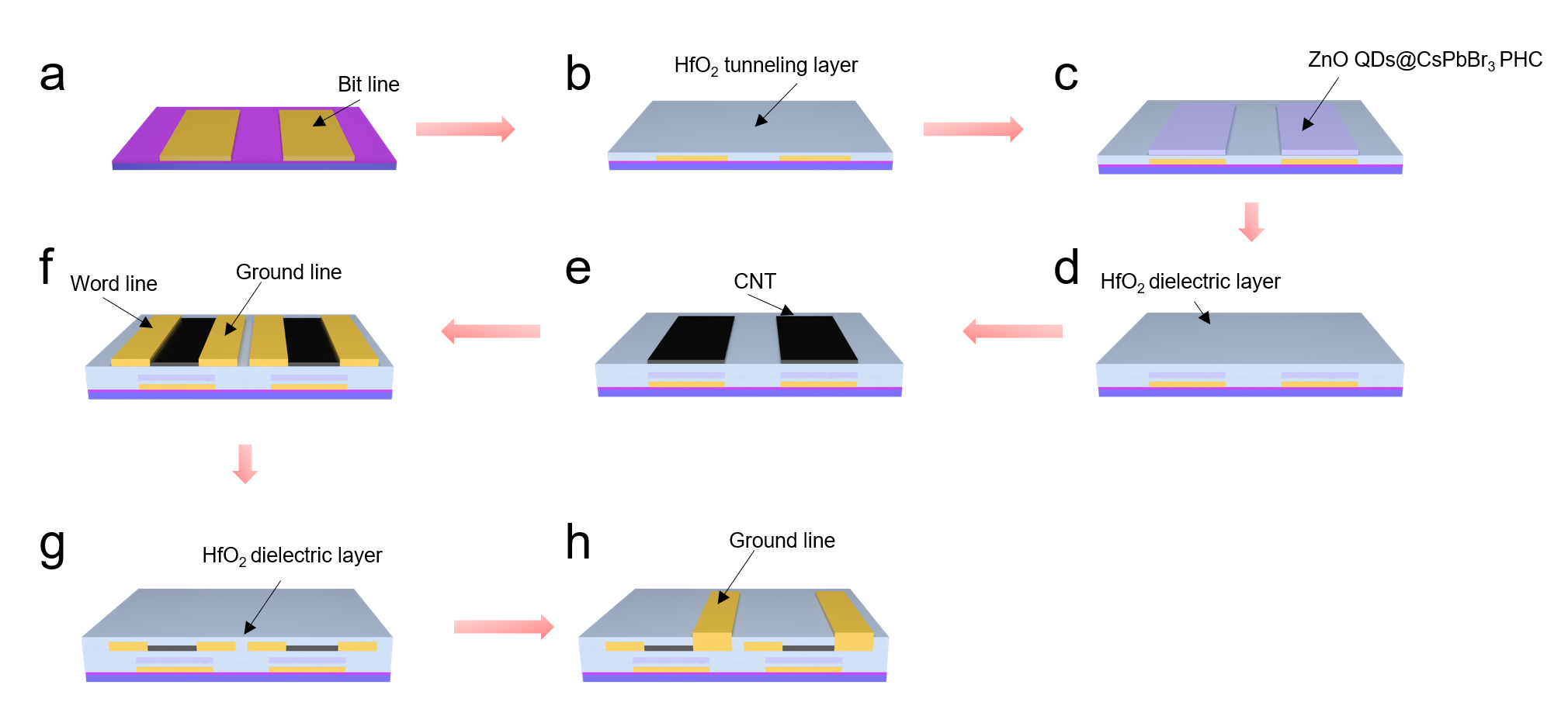


**Figure S25.** Schematic fabrication process of the FG-PT array. a) Electron beam deposition of gate electrodes (Ti/Au: 5/50 nm), which serve as bit lines. b) Atomic layer deposition of HfO_2_ tunneling layer. c) Spin coating of ZnO QDs@CsPbBr_3_ PHC film and patterning by photolithography and HCl solution etching. d) Atomic layer deposition of HfO_2_ dielectric layer. e) Deposition of CNT film and patterning by photolithography and oxygen plasma etching. f) Electron beam deposition of electrodes (Ti/Au: 5/50 nm) functioning as ‘word lines’ and ‘ground lines’, where the word line is connected to drain and ground line is connected to source. g) Atomic layer deposition of HfO_2_ dielectric layer to avoid crosstalk. h) HfO_2_ dielectric layer etching and electron beam deposition of Ti/Au(5/50 nm) lines to connect ‘ground lines’.


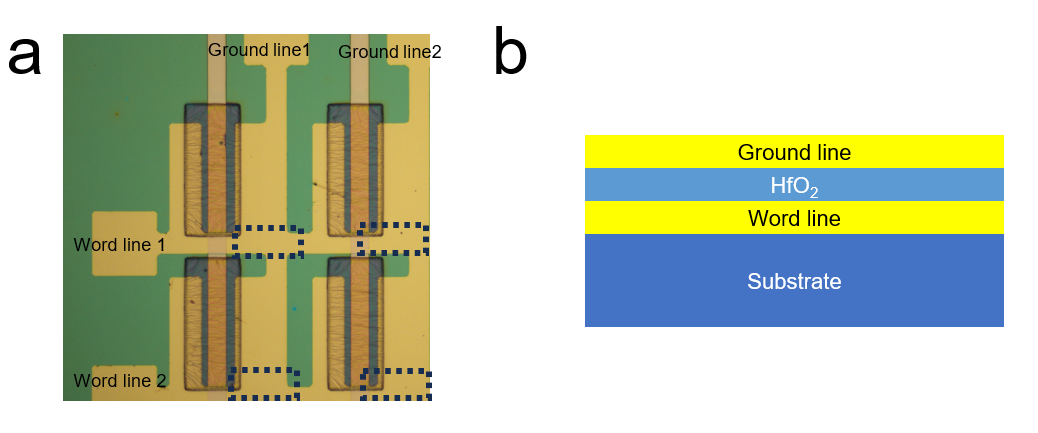


**Figure S26.** a) The enlarged view of boxed area in Figure 3i. b) Cross-sectional schematic diagram of the boxed area in (a).


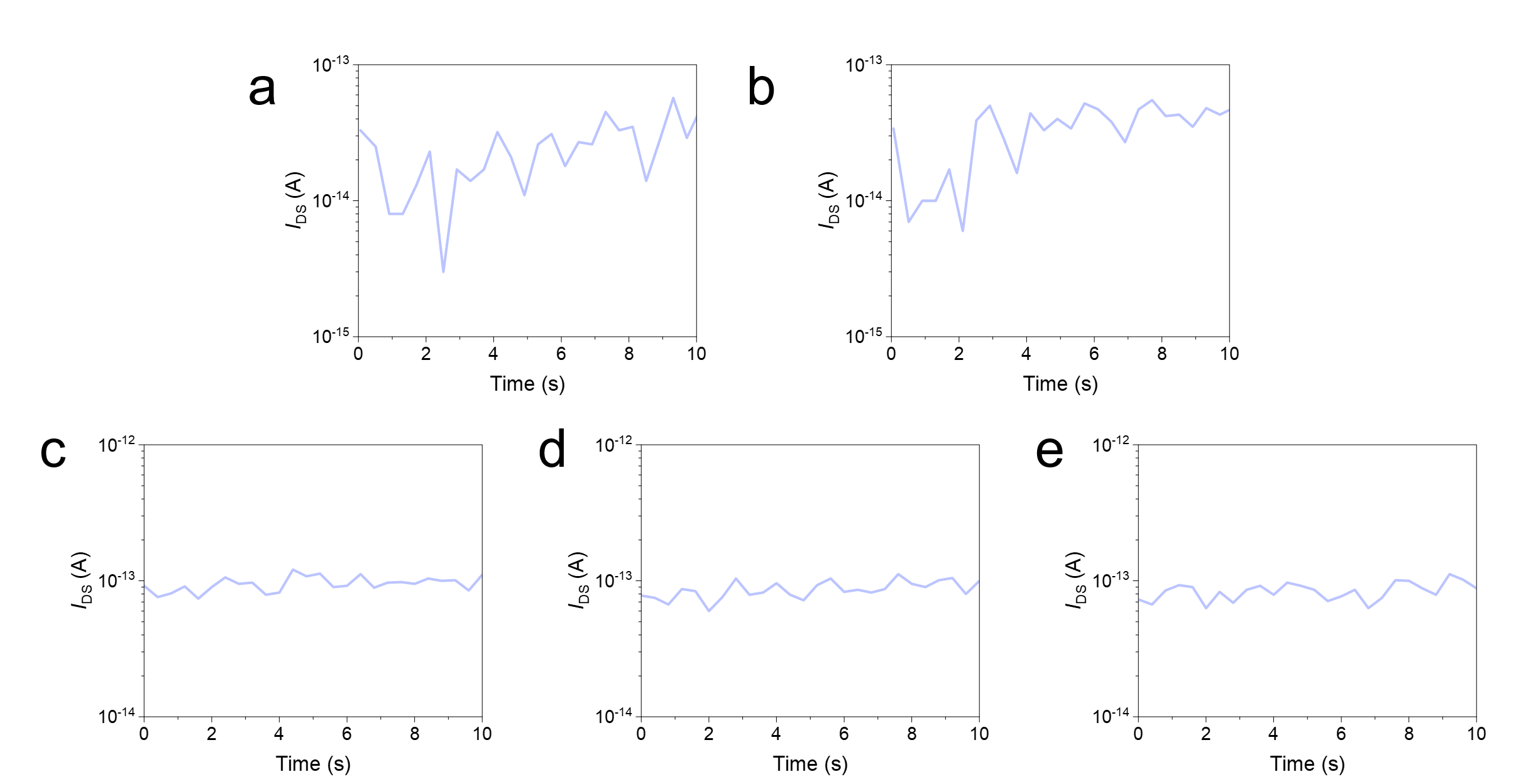


**Figure S27.** a) Leakage current of word line1 and word line2 of FG-PT array under 1 V. b) Leakage current of ground line1 and ground line2 of FG-PT array under 1 V. c) Leakage current of the (1,2) device during hole writing in the (1,1) device using *V*_GS_. Leakage current of the (1,2) device (d) and the (2,1) device (e) during hole erasure in the (1,1) device using light pulse.


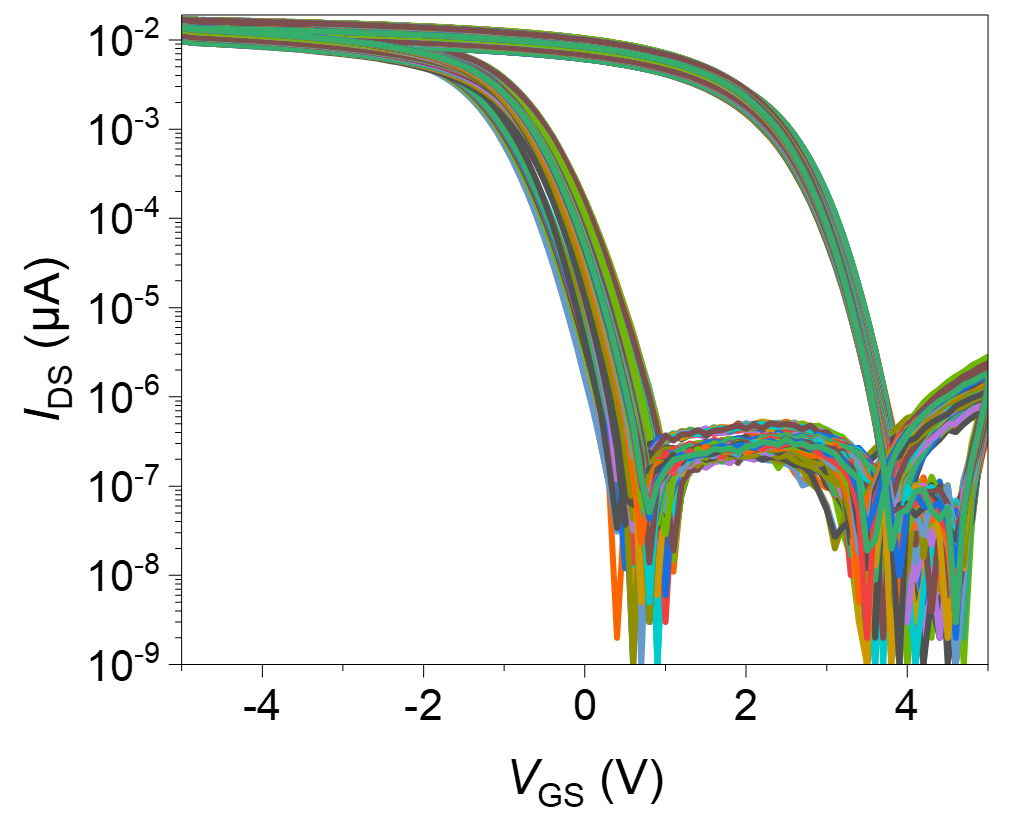


**Figure S28.** Transfer curves of different 256 devices in FG-PT array in the dark. *V*_DS_=100 mV.


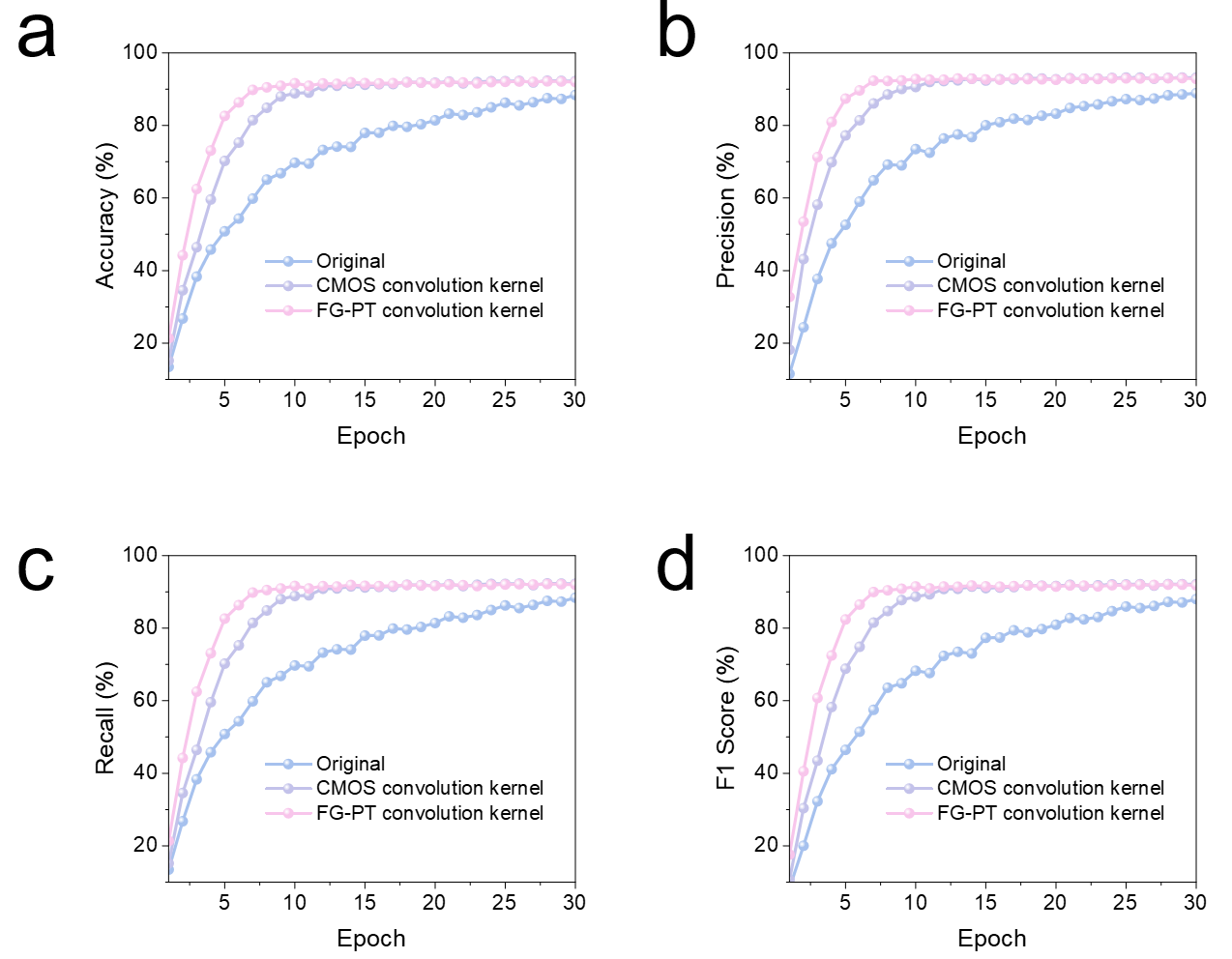


**Figure S29.** The variation of different parameters in the confusion matrix with the number of training epochs. a) Caculated accuracy, b) Precision, c) Recall and d) F1 score as a function of training epochs.

**
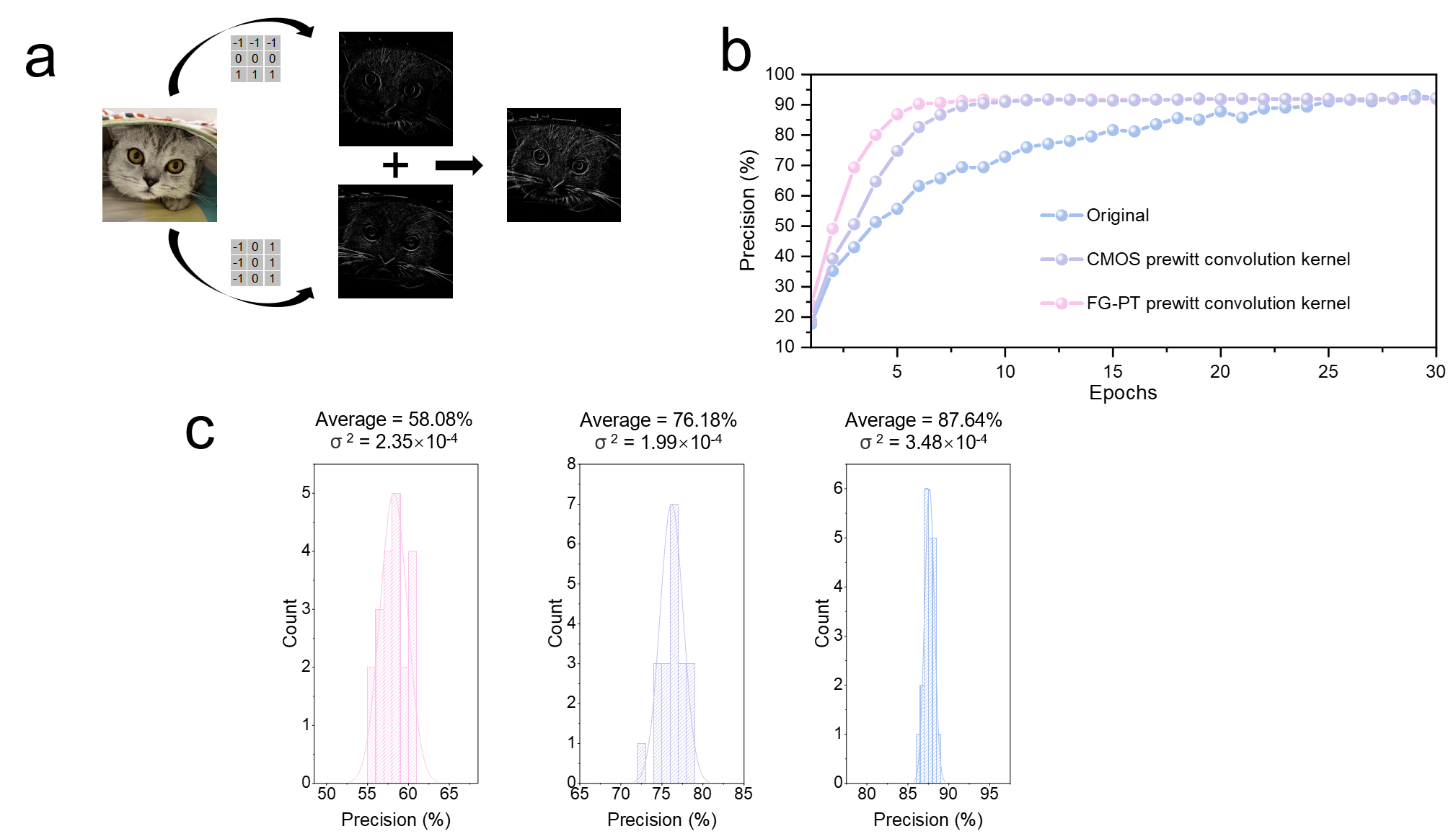
**

**Figure S30.** a) Schematic of image convolution processing with prewitt convolution kernel. b) The dependence of recognition precision on epochs with different convolution kernels. c) The precision distribution of images obtained by original images (left), CMOS convolution kernel processed images (middle) and FG-PT convolution kernel processed images (right) at the 5th epoch.

**Tables S1 to S3**

**Table S1.** TEM-EDS point analysis of ZnO QDs@CsPbBr_3_.

| **Element** | **Weight %** | **Atomic %** |
| --- | --- | --- |
| **O(K)** | 0.87 | 5.62 |
| **Zn(K)** | 1.50 | 2.36 |
| **Br(K)** | 55.59 | 71.24 |
| **Pb(L)** | 42.02 | 20.76 |

**Table S2.** XAFS fitting parameters at the Pb K-edge for various samples（Ѕ_0_^2^=0.84）

|  | **Shell** | **CN** | **R(Å)** | **Σ^2^** | **ΔE_0_** | **R factor** |
| --- | --- | --- | --- | --- | --- | --- |
| **PbO** | Pb-O | 6 | 2.16±0.01 | 0.0039 | -0.5±1.4 | 0.0031 |
| **CsPbBr_3_** | Pb-Br | 3.5±0.2 | 2.93±0.01 | 0.0106 | -5.6±2.1 | 0.0055 |
| **ZnO QDs@CsPbBr_3_** | Pb-Br | 5.1±0.2 | 2.95±0.01 | 0.0140 | -3.2±1.5 | 0.0031 |

The crystallographic parameters of PbO were obtained from the ICSD (Inorganic Crystal Structure Database).

*CN*: coordination numbers.

*R*: bond distance.

*Σ*^2^: Debye-Waller factors.

Δ*E*_0_: the inner potential correction.

*R* factor: goodness of fit.

*Ѕ*_0_^2^ was set to 0.84, according to the experimental XAFS fit of PbO reference by fixing CN as the known crystallographic value.

**Table S3.** Comparison of the floating gate materials, integration method, device number, area, conductance ratio, and operating voltage of our FG-PT with reported FG-PT at room temperature.

|  | **Floating gate materials** | **Integration method** | **Device number** | **Area (μm^2^)** | **Conductance ratio** | **Operating voltage (V)** | **Ref.** |
| --- | --- | --- | --- | --- | --- | --- | --- |
| **2D Material** | Mechanical exfoliated WSe_2_ | Common source/drain, EBL^a)^ | 9 | 1500 | 1.875 | 18 | 5 |
|  | Mechanical exfoliated Graphene | EBL | 1 | 40 | 1000 | 10 | 6 |
| **Insulator** | HfO_2_ via ALD ^b)^ | Isolated, EBL | 12 | 450 | 35 | 14 | 7 |
| **Organic** | Spin-coated CsPbBr_3_ QDs/  polystyrene  Composite | - | 1 | ~950 | 5000 | 200 | 8 |
|  | Spin-coated PbS QDs/PMMA | Isolated, Hard mask | 36 | ~10^8^ | 1000 | 10 | 9 |
| **Perovskite** | Mechanical exfoliated (BA)_2_MAPb_4_I_7_ | Hard mask | 1 | 400 | 16000 | 60 | 10 |
|  | CsPbBr_3_ via vapor deposition | Hard mask | 1 | 1000 | 52000 | 13 | 11 |
|  | Spin-coated CsPbBr_3_ | Hard mask | 1 | 1300 | 75 | 15 | 12 |
| **Our work** | Spin-coated ZnO QDs@  CsPbBr_3_ PHC | Common source/drain, photolithography | 25600 | 1.005×10^8^ | 357000 | 1 |  |

^a)^EBL: Electron Beam Lithography.

^b)^ALD: Atomic Layer Deposition.

**References**

1. C. Li, X. Chen, Z. Zhang, X. Wu, T. Yu, R. Bie, D. Yang, Y. Yao, Z. Wang, L. Sun, Charge-selective 2D heterointerface-driven multifunctional floating gate memory for in situ sensing-memory-computing, *Nano Lett.* **2024**, 24, 15025.
2. L. Wu, A. Wang, J. Shi, J. Yan, Z. Zhou, C. Bian, J. Ma, R. Ma, H. Liu, J. Chen, Y. Huang, W. Zhou, L. Bao, M. Ouyang, S. Pennycook, S. Pantelides, H. Gao, Atomically sharp interface enabled ultrahigh-speed non-volatile memory devices, *Nat. Nanotechnol.* **2021**, 16, 882.
3. J. Robertson, B. Falabretti, Band offsets of high K gate oxides on III-V semiconductors, *J. Appl. Phys.* **2006**, 100, 014111.
4. Y. Hinuma1, A. Grüneis, G. Kresse, F. Oba, Band alignment of semiconductors from density-functional theory and many-body perturbation theory, *Phys. Rev. B* **2014**, 90, 155405.
5. Z. Zhang, S. Wang, C. Liu, R. Xie, W. Hu , P. Zhou , All-in-one two-dimensional retinomorphic hardware device for motion detection and recognition, *Nat. Nanotechnol.* **2022**, 17, 27.
6. W. Li, T. Mu, Y. Chen, M. Dai, P. Sun, J. Li, W. Li, Z. Chen, Z. Wang, R. Yang, Z. Chen, Y. Wang, Y. Wu, S. Wang, Investigation of non-volatile and photoelectric storage characteristics for MoS_2_/h-BN/graphene heterojunction floating-gate transistor with the different tunneling layer thicknesses, *Micro Nanostruct.* **2024**, 187, 207764.
7. S. Lee, R. Peng, C. Wu, M. Li, Programmable black phosphorus image sensor for broadband optoelectronic edge computing, *Nat. Commun.* **2022**, 13, 1485.
8. R. Jin, J. Wang, K. Shi, B. Qiu, L. Ma, S. Huang, Z. Li, Multilevel storage and photoinduced-reset memory by an inorganic perovskite quantum-dot/polystyrene floating-gate organic transistor, *RSC Adv.* **2020**, 10, 43225.
9. J. Zhang, P. Guo, Z. Guo, L. Li, T. Sun, D. Liu, L. Tian, G. Zu, L. Xiong, J. Zhang, J. Huang, Retina-inspired artificial synapses with ultraviolet to near-infrared broadband responses for energy-efficient neuromorphic visual systems, *Adv. Funct. Mater.* **2023**, 33, 2302885.
10. H. Lai, Y. Zhou, H. Zhou, N. Zhang, X. Ding, P. Liu, X. Wang, W. Xie, Photoinduced multi-bit nonvolatile memory based on a van der waals heterostructure with a 2D-perovskite floating gate, *Adv. Mater.* **2022**, 34, 2110278.
11. J. Pei, X. Wu, W. Liu, D. Zhang, S. Ding, Photoelectric logic and in situ memory transistors with stepped floating gates of perovskite quantum dots, *ACS Nano* **2022**, 16, 2442.
12. Q. Li, T. Li, Y. Zhang, H. Zhao, J. Li, J. Yao, Dual-functional optoelectronic memories based on ternary hybrid floating gate layers, *Nanoscale* **2021**, 13, 3295.
